# NIR-II Nanomedicine Engineered CAR-NK Cells for Precision Navigation and Potentiating Lung Cancer Immunotherapy by Remodeling Tumor Microenvironment

**DOI:** 10.1021/jacs.6c01128

**Published:** 2026-03-06

**Authors:** Yeneng Dai, Qihang Ding, Ze Chen, Guanda Jiao, Yiqi Yang, Shengyu Fu, Ziyi Yang, Xiaoxi Liu, Kun Qian, Zhen Cheng, Dongliang Leng, Qi Zhao

**Affiliations:** † Cancer Centre, Institute of Translational Medicine, Faculty of Health Sciences, 59193 University of Macau, Taipa, Macau SAR 999078, China; ‡ MoE Frontiers Science Center for Precision Oncology, University of Macau, Taipa, Macau SAR 999078, China; § State Key Laboratory of Drug Research, Molecular Imaging Center, Shanghai Institute of Materia Medica, Chinese Academy of Sciences, Shanghai 201203, China; ∥ Shandong Laboratory of Yantai Drug Discovery, Bohai Rim Advanced Research Institute for Drug Discovery, Yantai, Shandong 264117, China; ⊥ Department of Chemistry, 34973 Korea University, Seoul 02841, Korea

## Abstract

Despite the prominent success against hematologic malignancies in clinical settings, chimeric antigen receptor NK (CAR-NK) cell immunotherapy is still hindered in lung cancer tumors owing to insufficient infiltration and poor immune activation induced by the immunosuppressive tumor microenvironment (TME). Herein, a nanoengineered CAR-NK biohybrid (CK-PSI) was constructed by conjugating nanomedicine (PSI NPs) containing a near-infrared II (NIR-II) polymer and a specific small-molecule inhibitor of Smad3 (SIS3) to the surface of metabolic glycan-engineered CAR-NK cells via a bioorthogonal reaction. Anti-B7H3 CAR modification on cell vectors offers selectively targeted delivery of hitchhiking NIR-II nanomedicine into lung cancer tumors, simultaneously enabling real-time tracking of CAR-NK cells and precise localization of deep-seated tumors through NIR-II fluorescence imaging. NIR-II excitation mild photothermal therapy not only destroys tumor cells by thermal ablation but also promotes the infiltration and penetration of CK-PSI through the rupture of physical barriers within tumor tissues. More importantly, the *in situ* release of the Smad3 inhibitor further reduces extracellular matrix (ECM) deposition through TGF-β signaling pathway blockade in the TME, thereby boosting the infiltration and immune activation of CAR-NK cells. The bioorthogonal nanohybrid with NIR-II phototheranostic triggered cell localization and immunomodulatory capabilities provides a new paradigm for potentiating the infiltration and immune activation efficiency of CAR-NK cells against solid tumors.

## Graphical abstract

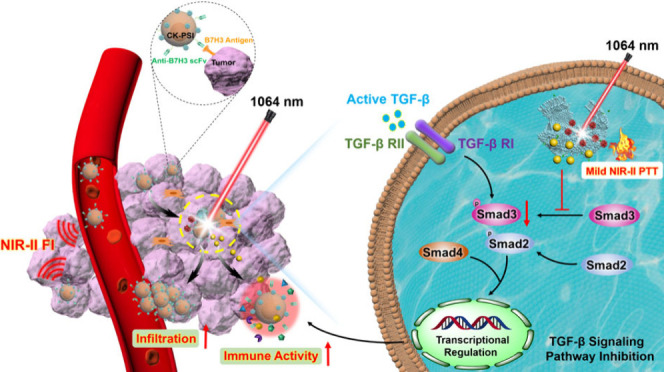

## Introduction

Natural killer (NK) cells, as innate lymphoid cells with easy access and expansion, are capable of spontaneously recognizing target cells and eliciting a powerful cytotoxicity accompanied by cytokine release to selectively kill aberrant cells without strict human leukocyte antigen (HLA) matching and without producing cytokine release syndromes (CRS).^1–4^ Chimeric antigen receptor (CAR)-engineered NK cell therapy (CAR-NK therapy) has brought substantial breakthroughs in many clinical trials to treat hematologic malignancies due to targeted immune surveillance, lower costs, and fewer toxic side effects.^5–7^ With considerable ongoing efforts on developing CAR-NK cell-based immunotherapies, it is of increasing importance to track and locate these adaptive immune cells *in viv*o, which can reflect the retention and activation status of immune cells, providing crucial information for precise intervention, on-target activation, and safety design.^8–10^ Imaging-based cell tracking techniques have been developed for real-time localization and activation effect evaluation of immune cells, including ultrasound imaging,^11^ magnetic resonance imaging,^12^ and optical imaging.^13^ Among these imaging modalities, fluorescence imaging (FI) possesses significant advantages with regard to high sensitivity and nonionizing radiation.^14^ In particular, FI in the second near-infrared (NIR-II, 1000–1700 nm) window holds greater advantages over that in the NIR-I (700–900 nm) window due to the deeper tissue penetration depth and reduced photon scattering, contributing new insights of systemic distribution and monitoring of immune cells.^15–17^ Moreover, NIR-II photothermal therapy (PTT) enables thermal ablation of deep-seated tumors with much higher maximum permissible exposure (MPE).^18,19^ However, NIR-II fluorescence mediated real-time localization of CAR-NK cells has not been widely explored considering the complex cell modification procedures and cumbersome NIR-II fluorescent probe design and synthesis.

Despite the encouraging success, the immune output of CAR-NK cell therapy in solid tumors, especially in lung cancer tumors, still faces considerable challenges owing to immunosuppressive tumor microenvironment (TME).^20,21^ The rapidly proliferative malignant tumor cells overexpress transforming growth factor-β (TGF-β), which causes abnormally activated cancer-associated fibroblasts (CAFs) and uncontrolled extracellular matrix (ECM) deposition through the downstream Smad2/3 protein mediated signaling pathway.^22–24^ The dense cross-links between tumor tissue and ECM form a physical barrier with compressed vasculature and severe hypoxia.^25,26^ This would lead to insufficient homing and infiltration of CAR-NK cells into solid tumors.^27^ Moreover, the TGF-β signaling pathway can blunt the antitumor activity of CAR-NK cells as well as the inhibition of cytokine production,^28–30^ leading to an augmented immune resistance and immune escape. SIS3, a selective Smad3 inhibitor, has been proven to not only breach the physical barrier as an antifibrotic drug by Smad3-phosphorylation (p-Smad3) inhibition but also effectively promote the immune activity and proliferation of NK cells.^31–33^ However, their efficiency is still impeded due to low water solubility and toxic side effects to normal organs induced by nonspecific accumulation.^34,35^ Therefore, an alternative approach is desired for the targeted delivery of SIS3 and boosting the infiltration and immune activity of CAR-NK cells by overcoming the physical barriers of solid tumors.

In view of the above challenges, we designed and prepared a targeted biohybrid with hitchhiking delivery capability through bioorthogonal modification for boosting the efficacy of CAR-NK cell immunotherapy against lung cancer tumors. Semiconducting polymers with excellent NIR-II FI capability were first synthesized through a donor doping strategy. Then NIR-II nanomedicine was prepared by coencapsulation of NIR-II polymers and Smad3 inhibitor SIS3 into thermosensitive liposomes with dibenzocyclooctyl (DBCO) modification to form polymer-SIS3 nanoparticles (PSI NPs), followed by conjugation to the surface of metabolic glycan-engineered CAR-NK cells through bioorthogonal reaction, obtaining CAR-NK-PSI biohybrids (CK-PSI) (Scheme 1). Anti-B7H3 CAR modification endows CK-PSI with targeted delivery of NIR-II nanomedicine into solid tumors by positively binding to lung tumor cells overexpressing B7H3 antigen. The hitchhiking PSI NPs cargo enabled NIR-II FI of CAR-NK cells and solid tumors during targeted delivery, providing real-time monitoring of CAR-NK cells and precise localization of deep-seated tumors. NIR-II laser excitation mild photothermal effects not only ablated tumor cells but also enhanced the infiltration and penetration of CK-PSI by destroying dense ECM, accompanied by tumor vascular dilation and hypoxia relief. More importantly, the released SIS3 inhibited Smad3-phosphorylation to block the TGF-β signaling pathway, further reducing ECM generation and enhancing antitumor immune activity of CAR-NK cells. This living cell nanohybrid with integration of targeted nanomedicine delivery and NIR-II phototheranostic triggered immune activation into a single therapeutic platform provides a new strategy for potentiating efficiency of adoptive immunotherapy.

**Scheme 1:**
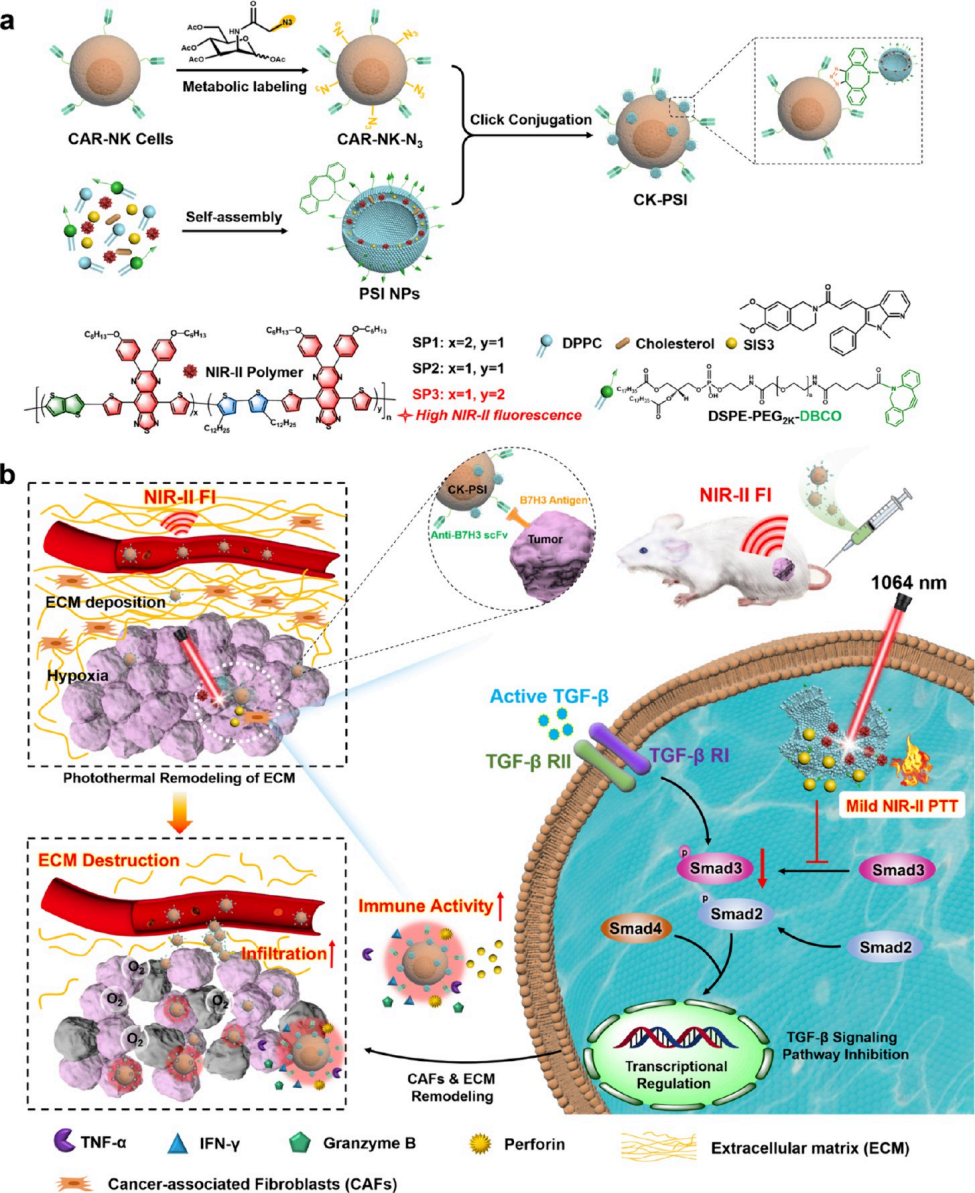
Schematic Illustration of CK-PSI Biohybrids for Potentiating Immunotherapy through Tumor Microenvironment (TME) Remodeling^a^

a (a) Preparation of CK-PSI biohybrids via bioorthogonal reactions. (b) NIR-II fluorescence imaging guided boosted infiltration and immune activation of CK-PSI towards lung cancer tumors through the synergistic effects of mild NIR-II photothermal intervention and TGF-β signaling blockade.

## Results and Discussion

### Etiological Analysis of Fibrotic Lung Cancer

To explore the fibrotic mechanism of lung cancer, the ECM levels of lung cancer patients in clinical practice were analyzed by using the cancer genome atlas (TCGA) databases. Bioinformatics analysis revealed a remarkable upregulation of the ECM marker collagen I in lung cancer tumors compared with normal lung tissues (Figure 1a). This would lead to dense tumor interstitial pressure with vasculature constriction and severe hypoxia, forming a physical barrier for drug penetration and immune cell infiltration. In particular, the α smooth muscle actin (α-SMA) protein, encoded by the ACTA2 gene, plays a critical role in the CAFs formation process. As revealed in Figure 1b and Figure 1c, the expression levels of ECM protein fibronectin and collagen I were positively correlated with the expression of the ACTA2 gene, respectively. This confirms that the activation of CAFs in lung cancer promotes the expression of ECM proteins, ultimately inducing dense ECM deposition, which provides a universal reason for the poor immune output of CAR-NK cell therapy against lung cancer tumors.

**Figure 1:**
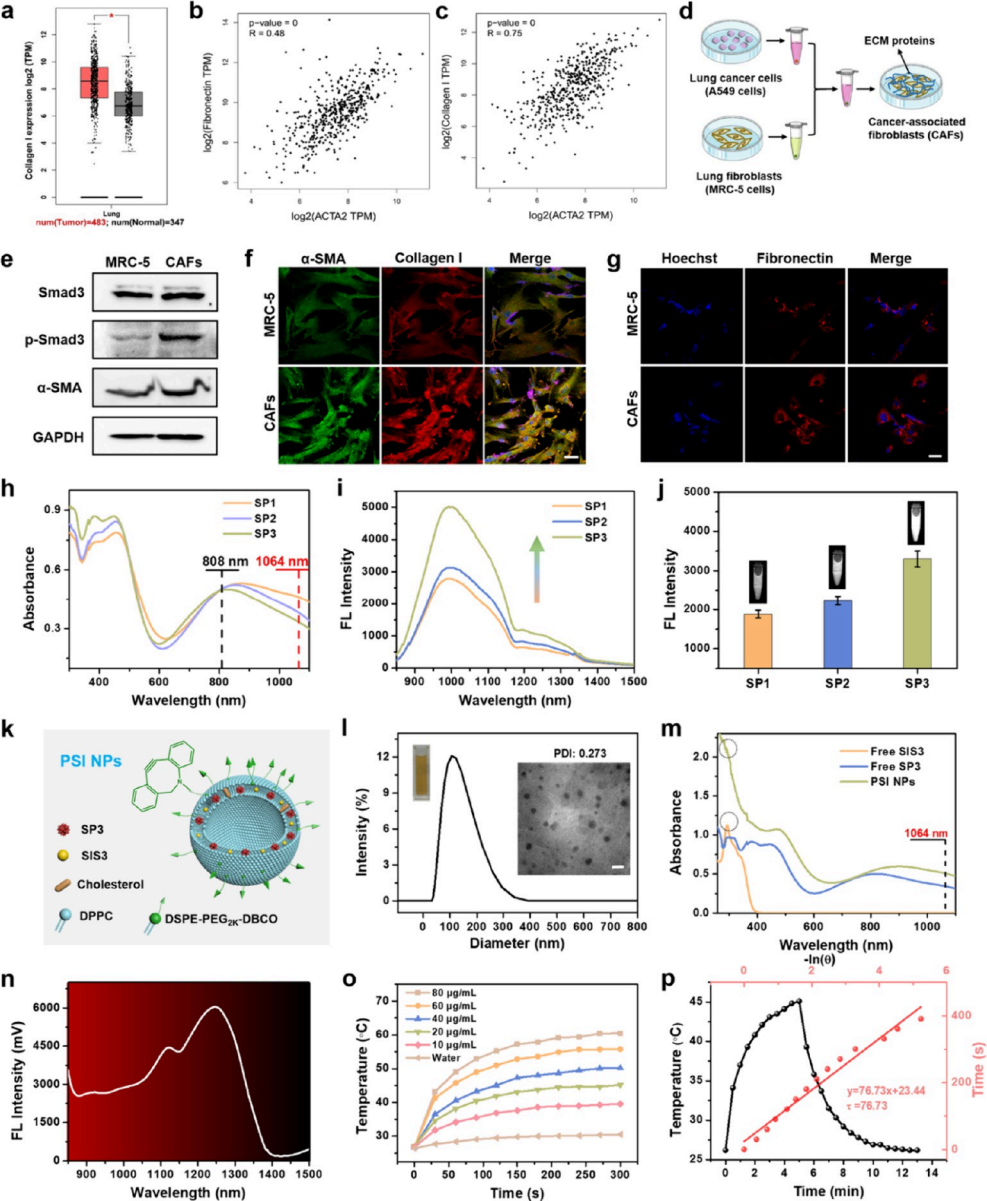
Etiological analysis of fibrotic lung cancer and preparation and characterization of NIR-II nanomedicine. (a) The expression difference of collagen I in lung cancer (*n* = 483) and normal lung tissue (*n* = 347) from TCGA lung cancer database. The correlation between the levels of (b) fibronectin and (c) collagen I with the expression of ACTA2 gene. R > 0 represents a positive correlation. (d) Schematic diagram of CAFs generation. (e) Western blot analysis of Smad3, p-Smad3, and α-SMA protein expression in MRC-5 cells and CAFs. Immunofluorescence images of (f) α-SMA and collagen I protein together with (g) fibronectin protein in MRC-5 cells and CAFs. Scale bar: 50 μm. (h) UV–vis absorption spectra of SPs (SP1, SP2, SP3) in THF with the same 808 nm absorption and (i) corresponding fluorescence emission spectra under 808 nm excitation. (j) NIR-II fluorescence images (1075 nm long-pass filter) and quantified mean intensity. (k) Schematic diagram for the composition of PSI NPs. (l) TEM image and hydrodynamic diameter of PSI NPs. Scale bar: 200 nm. (m) Absorption and (n) fluorescence emission spectra of PSI NPs. (o) Temperature changes and (p) photothermal conversion efficiency calculation of PSI NPs aqueous solution under 1064 nm laser irradiation (1 W/cm^2^).

According to previously reported literature,^31^ TGF-β secreted from tumor cells plays a promoting role in the CAFs activation and ECM deposition through the TGF-β/Smad3 signaling pathway. To verify the mechanism of lung cancer tumor-induced fibrosis, MRC-5 cells, a human embryonic lung fibroblasts cell line, were cultured with conditioned medium from lung cancer to induce CAFs generation (Figure 1d). Western blot analysis demonstrated the transformation of MRC-5 cells into CAFs through the significantly elevated expression of Smad3-phosphorylation and α-SMA protein (Figure 1e), evidenced by brighter immunofluorescence of α-SMA in CAFs compared with MRC-5 cells (Figure 1f). Immunofluorescence analysis further substantiated the remarkably increased expression of ECM proteins in CAFs, including fibronectin and collagen I (Figure 1f and Figure 1g). These results confirm that TGF-β secreted by lung cancer tumors promotes CAFs activation and ECM deposition through the TGF-β/Smad3 signaling pathway. Therefore, it may be an effective strategy to inhibit the TGF-β signaling pathway in CAFs, thereby breaching the physical barrier to enhance the infiltration of CAR-NK cells and restore immune activity toward lung cancer.

### Synthesis and Characterization of NIR-II Semiconducting Polymers

In order to obtain phototherapeutic agents with high NIR-II fluorescence performances, Stille coupling polymerization was performed for synthesis of semiconducting polymers (SP1, SP2, SP3) by adjusting the donor doping ratios (Scheme S1), using long alkyl side chain-modified bithiophene (TC) and planar 2,5-bis­(trimethylstannyl) thieno [3,2-*b*]­thiophene (DT) as donor units as well as electron-deficient (4,9-bis­(5-bromothiophen-2-yl)-6,7-bis­(4-(hexyloxy)­phenyl)-[1,2,5]­thiadiazolo­[3,4-g]­quinoxaline (TTQ) as acceptor units. TTQ with strong electron-withdrawing ability and π-conjugation can lead to long-wavelength absorption/emission.^36^ The planarity of the electron donor DT ensures the high absorption extinction of the polymer through π-π interaction, and TC with long alkyl side chains is expected to afford high fluorescence intensity through steric hindrance caused by the twisted structure.^37,38^ The structures of the synthesized SPs were verified by ^1^H NMR (Figures S1–S3). The significant hydrogen signals from the alkyl side chains were observed in the range of 0.5 to 2.0 ppm. The hydrogen signals of thiophene in the polymer structure were shown in the range of 7.5 to 8.2 ppm. Gel permeation chromatography (GPC) revealed that the number-average molecular weights (*M*_n_) of SP1, SP2, and SP3 were 58,583, 56,600, and 48,165, respectively, indicating the successful synthesis of polymers (Table S1). The number-average polymerization degrees of SP1, SP2, and SP3 are calculated to be approximately 20.09, 27.28, and 14.69, respectively. The photophysical properties were investigated in the conventional organic solvent tetrahydrofuran (THF). All the SPs showed broad absorption bands in the near-infrared region with a significant absorption at 1064 nm (Figure 1h), which was confirmed by the narrow band gaps between the molecular orbitals by density functional theory (DFT) calculations (Figure S4). As TC doping ratios increased in the polymer backbone, a slight blue shift of the absorption band was observed. All the SPs exhibited distinct NIR-II fluorescence emission with an emission peak at approximately 993 nm (Figure 1i). It is noteworthy that the fluorescence intensity of the SPs gradually increased with an increased level of TC doping when the absorption was fixed to the same value at 808 nm. In particular, SP3 exhibited prominent NIR-II fluorescence emission, with approximately 1.80-fold and 1.61-fold enhancement over SP1 and SP2, respectively. Correspondingly, SP3 exhibited the brightest NIR-II fluorescence image at the same 808 nm optical absorption (Figure 1j). Similar fluorescence enhancement and imaging boosting trends were also verified at the same mass concentration (Figure S5 and Figure S6). This is possibly due to the increased intramolecular steric hindrance induced by the branched side chains of TC facilitating the radiative transition of SPs, thus resulting in the fluorescence enhancement of SP3.^38,39^ The larger dihedral angle between the TC and TTQ fragments, relative to that between DT and TTQ, displayed a more twisted structure of SP3 (Figure S7), which will contribute to the enhanced radiative decay to produce fluorescence. These results confirm that the increased level of TC doping endows SP3 with great potential for NIR-II FI *in vivo*.

### Preparation and Characterization of NIR-II Nanomedicine (PSI NPs)

Considering the superior NIR-II fluorescence imaging performance, SP3 was encapsulated into dibenzocyclooctyl (DBCO)-modified thermosensitive liposomes together with Smad3 inhibitor SIS3 through thin film hydration to prepare a NIR-II lipid nanomedicine (Polymer-SIS3 nanoparticles, PSI NPs) (Figure 1k). Transmission electron microscope (TEM) images showed uniformly dispersed spherical morphology of PSI NPs (Figure 1l). The hydrodynamic diameter of the NPs was determined to be approximately 106 nm by dynamic light scattering (DLS) analysis. The good dispersibility of the light brown solution was confirmed by a polydispersity index (PDI) of 0.273 and a zeta potential of approximately −16.1 mV (Figure S8). The PSI NPs aqueous solution exhibited a characteristic absorption peak of SIS3 at 294 nm (Figure 1m), indicating the successful loading of SIS3. Moreover, the introduction of NIR-II polymers endowed PSI NPs with a broad absorption range in both NIR-I and NIR-II regions. A strong absorption was observed at 1064 nm with a mass extinction coefficient of 12.2 L/g cm^–1^ (Figure S9), allowing for mild photothermal mediated TME remodeling triggered by NIR-II light. After liposome encapsulation, PSI NPs displayed a broad fluorescence emission ranging from 850 to 1400 nm under 808 nm excitation (Figure 1n), with a significantly red-shifted fluorescence emission peak at 1246 nm. This may be due to the increased intramolecular steric hindrance induced by the electron donor unit TC with long alkyl side chains promoting the twisted aggregation of SP3 within PSI NPs,^40,41^ making it a promising candidate for precise location of deep-seated tumors and real-time tracking of CAR-NK cells via NIR-II FI. NIR-II FI of PSI NPs revealed a positive linearity between the fluorescence intensity and concentrations, evidenced by the gradually brightening fluorescence images (Figure S10). These results verify the great potential of PSI NPs for the *in vivo* tracking of CAR-NK cells and NIR-II phototheranostics of deep-seated tumors.

The NIR-II photothermal effects of PSI NPs were investigated under continuous 1064 nm laser irradiation (1 W/cm^2^, 5 min). As shown in Figure 1o, the temperature of the PSI NPs aqueous solution gradually increased with the polymer concentration and irradiation time, as evidenced by the gradually enhanced photothermal images (Figure S11). At a concentration of 20 μg/mL, the final solution temperature could reach 45.2 °C, which was beneficial for mild photothermal-mediated tumor ablation and TME modulation. Distinct temperature differences were detected when the laser power density was adjusted (Figure S12a). In addition, the negligible temperature changes revealed the photothermal stability of the nanomedicine during continuous laser on/off cycles (Figure S12b). The photothermal conversion efficiency of PSI NPs was calculated as about 31.63% by one heating and cooling cycle (Figure 1p). These results suggest that PSI NPs can be used as NIR-II photothermal agents for the thermal ablation and TME remodeling of solid tumors. The photothermally induced release behavior of SIS3 from thermosensitive liposomes was investigated. NIR-II laser irradiation-induced photothermal effect promoted the release of SIS3 from PSI NPs, with a drug release of approximately 52.21% during sustained laser on–off cycling compared to the control group without laser irradiation (Figure S13).

### Generation of CAR-NK Cells and Preparation of CK-PSI Biohybrids

To obtain immune cells with targeting ability against human nonsmall cell lung cancer cell (NSCLC) line A549 cells overexpressing B7H3 antigen, according to our previous works,^42^ we constructed NK cells expressing a CAR construct containing anti-B7H3 scFv, the CD8 TM region and the intracellular domains of 4-1BB and CD3ζ (Figure 2a). Human CAR-NK cells were prepared by transduction of human NK-92 cells utilizing lentiviral particles with the coexpression of a green fluorescence protein Zs-Green. Almost all NK-92 cells showed green fluorescence of Zs-Green after transduction (Figure 2b). Flow cytometry analysis revealed a high expression rate of anti-B7H3 scFv on the surface of NK cells (Figure 2c), indicating the successful construction of CAR-NK cells. To obtain CAR-NK cell biohybrids, azide groups were first modified on the CAR-NK cell surface to obtain CAR-NK-N_3_ by intrinsic metabolic labeling of azido sugars. DBCO-modified PSI NPs were coincubated with CAR-NK-N_3_ to obtain CK-PSI biohybrids through bioorthogonal reaction (Figure 2a). Scanning electron microscopy (SEM) images showed that a large number of PSI NPs with the size of approximately 100 nm dispersed on the surface of CAR-NK cells in the CK-PSI group, indicating the successful conjugation of PSI NPs to CAR-NK cells (Figure 2d). After bioorthogonal conjugation, CK-PSI retained high binding toward the B7H3 antigen and did not compromise the targeting specificity of CAR-NK cells (Figure S14). DBCO-modified PSI NPs encapsulating DiR were coincubated with CAR-NK-N_3_ for visualization of the click reaction. As shown in Figure 2e, the bright red fluorescence of DiR was observed on the surface of CAR-NK cells, implying the successful attachment of PSI NPs. Notably, azide modification could result in more retention of PSI NPs on the CAR-NK cell surface compared with the unmodified group through enhanced red fluorescence signals (Figure 2f), confirmed by a higher positive cell population of about 94.6% by flow cytometry analysis (Figure 2g). Concentration-dependent conjugation toward CAR-NK cells was observed in Figure S15. Interestingly, CK-PSI biohybrids successfully inherited the photophysical properties of PSI NPs, displaying significant absorption and fluorescence emission in the NIR-II region, as well as an emission peak at approximately 1250 nm (Figure S16 and Figure 2h). CK-PSI solution exhibited concentration-dependent NIR-II fluorescence signals under 808 nm excitation (Figure S17). These results demonstrate the potential application of CK-PSI for targeted drug delivery and NIR-II phototheranostics via a hitchhiking strategy.

**Figure 2:**
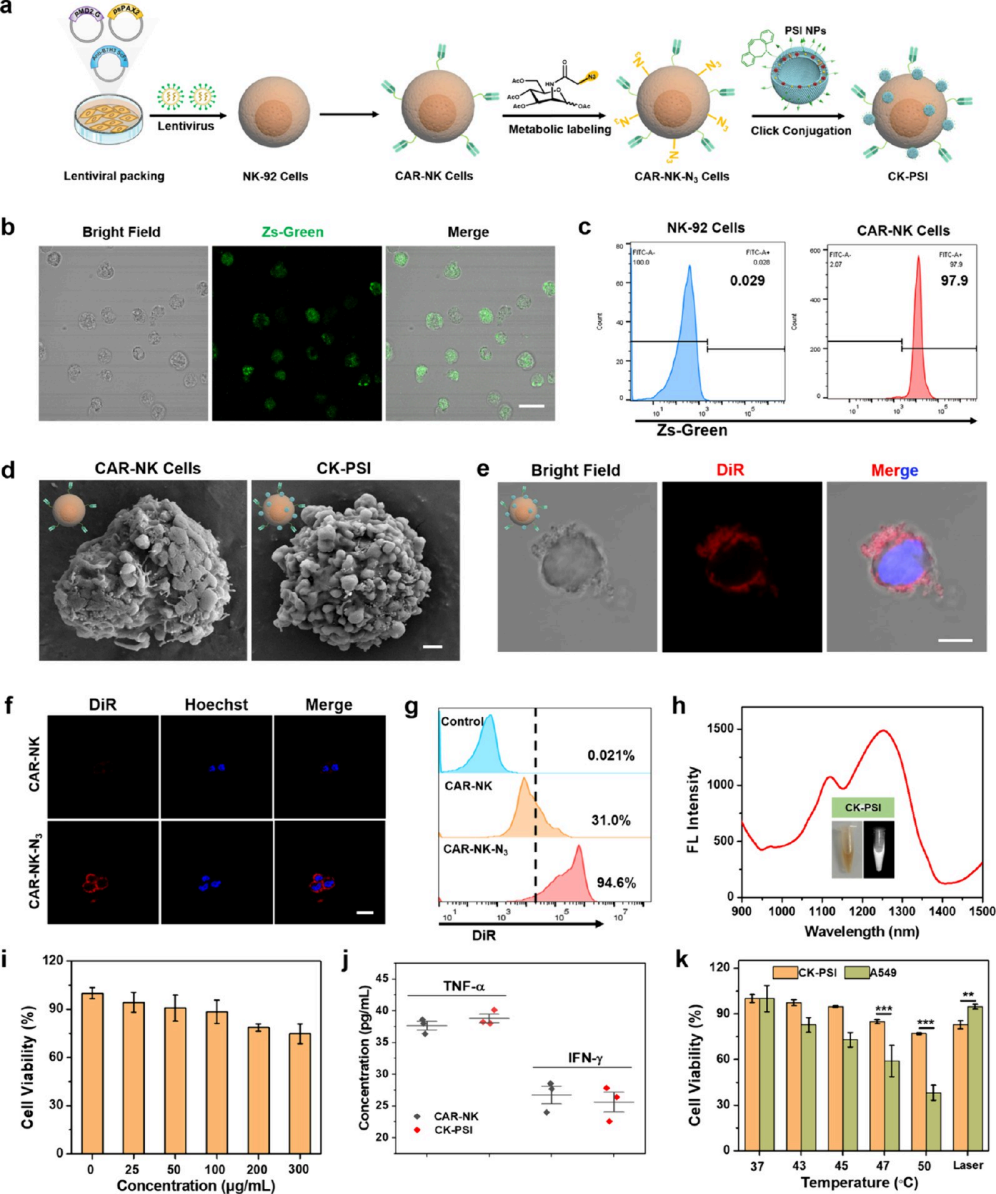
Preparation and characterization of CK-PSI biohybrids. (a) Schematic diagram of CAR-NK cell generation and CK-PSI preparation. (b) Fluorescence images of NK-92 cells expressing anti-B7H3 scFv. Scale bar: 20 μm. (c) Generation ratios of CAR-NK cells by flow cytometry. (d) SEM images of CAR-NK cells and CK-PSI. Scale bar: 1.0 μm. (e) Fluorescence images of CK-PSI containing fluorescent dye DiR. Scale bar: 10 μm. (f) Fluorescence images and (g) flow cytometric analysis of CAR-NK cells with or without azide modification after bioorthogonal conjugation. Scale bar: 20 μm. (h) The fluorescence spectrum of CK-PSI and corresponding NIR-II fluorescence images. (i) Cell viability of CK-PSI with different concentrations of PSI NPs. (j) Cytokine release of CAR-NK cells before and after bioorthogonal conjugation. (k) Cell viability of A549 cells and CK-PSI biohybrids at different incubation temperatures. **p < 0.01 and ***p < 0.001.

### Cell Activity and Thermotolerance Assay of CK-PSI Biohybrids

To investigate the influence of bioorthogonal reaction on the activity of CAR-NK cells, PSI NPs with various concentrations were added to the conjugation reaction, and the cell viability of CK-PSI cells was tested by cell counting kit-8 (CCK-8) assay. The cell viability of CK-PSI could still reach 74.67% even at the high PSI NPs concentration (Figure 2i). In addition, CK-PSI exhibited similar cytokine (TNF-α and IFN-γ) release ability compared with nonconjugated CAR-NK cells at a conjugation concentration of 300 μg/mL (Figure 2j). Taken together, this demonstrates that bioorthogonal conjugation effectively preserved the viability and antitumor immune activity of CAR-NK cells. The thermotolerance of CK-PSI was further evaluated at different incubation temperatures. When the temperature was increased to 45 °C, CK-PSI retained cell viability of more than 90%. However, the survival rates of thermosensitive A549 cells drastically decreased to 72.95%, indicating thermal ablation-mediated tumor cell apoptosis (Figure 2k). To investigate the mechanism of the thermotolerance difference between A549 cells and CK-PSI, the expression of heat shock proteins (HSP90) responsible for heat resistance in the cells was examined. As shown in Figure S18, after mild heating and laser irradiation, HSP90 expression in CK-PSI was significantly elevated, with a 3.49-fold and 3.80-fold increase, respectively. However, HSP90 expression in A549 cells increased only by approximately 1.41-fold after mild heating. This indicates that CK-PSI can produce stronger heat resistance after heat stimulation compared with tumor cells. These results confirm that tumor cells can be severely damaged while CK-PSI exhibits good thermal resistance with negligible changes of biological functions under mild heating treatment.

### CAFs Reprogramming, Tumor Spheroid Infiltration, and Immune Activation Analysis *In Vitro*

Effective cell therapy requires augmented infiltration of CAR-NK cells into solid tumors through reshaping of the immunosuppressive TME. TGF-β overexpression during tumor proliferation attenuates the antitumor immune activity of CAR-NK cells as well as the limited secretion of cytokines by inducing ECM generation. Therefore, it is necessary to block the TGF-β signaling pathway to increase the efficiency of immunotherapy. MRC-5 cells were incubated with conditioned medium from lung cancer A549 cells, followed by treatment with CAR-NK cells, PSI NPs, and CK-PSI with or without 1064 nm laser irradiation (1 W/cm^2^) for 5 min, and CAFs reprogramming was evaluated (Figure 3a). The levels of the downstream phosphorylated Smad3 protein were detected by Western blot analysis. SIS3, PSI NPs, and CK-PSI treatment resulted in a significant decrease in p-Smad3 levels (Figure 3b and Figure S19). CK-PSI + Laser further inhibited the phosphorylation of Smad3, with an approximately 5.19-fold decrease compared with the control group (Figure 3c). As expected, the CAFs treated with PSI NPs and CK-PSI exhibited significantly decreased fluorescence signals of α-SMA and collagen I protein compared to the control group (Figure 3d), indicating the downregulation of ECM protein levels. Moreover, CAFs activation was further inhibited through CK-PSI plus NIR-II laser irradiation treatment, in which mean fluorescence intensity of α-SMA and collagen I protein decreased by approximately 1.65-fold and 1.59-fold relative to the CK-PSI group, respectively (Figure 3f and Figure 3g). This may be ascribed to the NIR-II photothermal triggered improved SIS3 release from thermosensitive liposome hitchhiking on CAR-NK cells.

**Figure 3:**
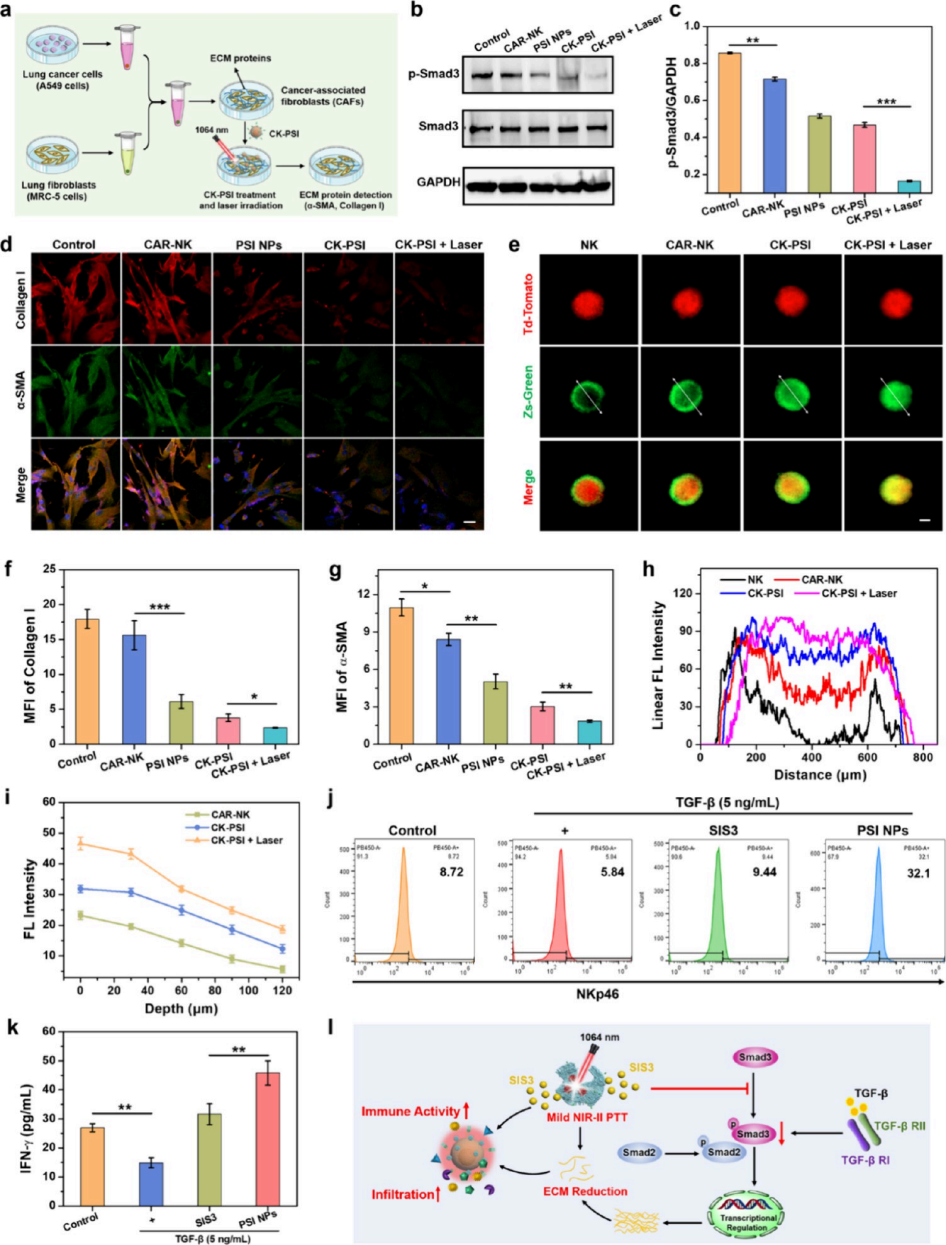
CAFs reprogramming, tumor infiltration, and immune activation. (a) Schematic illustration of CK-PSI mediated CAFs remodeling *in vitro*. (b) Western blot assay and (c) determination of p-Smad3 protein expression in CAFs. (d) Immunofluorescence images and (f, g) quantitative fluorescence analysis of α-SMA and collagen I protein in CAFs with various treatments. Scale bar: 50 μm. (e) Fluorescence images of A549 3D tumor spheroids expressing Td-Tomato with various treatments. Scale bar: 200 μm. (h) Fluorescence intensity analysis of marked regions in (e). (i) Fluorescence intensity of Zs-Green at different depths within 3D tumor spheroids. (j) Expression of NKp46 on the surface of CAR-NK cells and (k) IFN-γ secretion after different treatments. (l) Schematic illustration of CK-PSI mediated enhanced infiltration and amplified immune response. *p < 0.05, **p < 0.01, and ***p < 0.001.

To explore the tumor infiltration ability of CK-PSI, mixed 3D tumor spheroids of MRC-5 cells and A549 cells expressing Td-Tomato were incubated with Zs-Green modified NK cells, CAR-NK cells, and CK-PSI for 48 h, respectively, with or without 1064 nm laser irradiation, followed by observation under confocal microscopy. As displayed in Figure 3e, the green fluorescence ring in the NK cell group indicated that NK cells mainly accumulate at the edge of the cell spheroid due to penetrative obstruction and targeting lack. However, CAR-NK cells could be more enriched in the interior region of the tumor spheroid by dispersed green fluorescence, ascribed to the active targeting recognition toward A549 cells by CAR modified on the surface of NK cells. CK-PSI showed higher penetration toward tumor spheroids, possibly due to reduced CAFs activation and ECM deposition mediated by SIS3 mediated TGF-β signaling inhibition. Remarkably, CK-PSI plus laser irradiation exhibited the most intensive fluorescence throughout the cell spheroid (Figure 3h), suggesting that mild NIR-II photothermal intervention and TGF-β signaling inhibition could synergistically promote penetration and infiltration of CK-PSI in tumor cell spheroids. To further evaluate the cell penetration ability, the fluorescence signals of CAR-NK cells at different penetration depths within 3D tumor spheroids were detected with or without NIR-II laser irradiation. The CK-PSI plus laser irradiation group exhibited significantly higher green fluorescence signal than the other groups at various depths (Figure S20), with a 3.31-fold and 1.53-fold enhancement of fluorescence intensity over the CAR-NK group and CK-PSI group even at the depth of 120 μm, respectively (Figure 3i). These results demonstrate the improved penetration and infiltration of CK-PSI in the 3D tumor spheroids models under the aid of TGF-β signaling inhibition and mild NIR-II photothermal effect.

It has been proven that SIS3 enables higher expression of the activation receptor NKp46 on the surface of NK cells through TGF-β signaling blockade.^32,43^ To explore the reversal of SIS3 on the immune activity of CAR-NK cells, SIS3 and PSI NPs were incubated with CAR-NK cells in the presence of TGF-β. TGF-β treatment could downregulate NKp46 expression on CAR-NK cells compared to the control group, indicating the suppression of immune function (Figure 3j). Notably, SIS3 and PSI NPs treatment remarkably reversed the suppression of NKp46 induced by TGF-β. Correspondingly, the enhancement of NKp46 levels further promoted the secretion of IFN-γ from CAR-NK cells (Figure 3k). These results confirmed that CK-PSI biohybrids could evoke an immune self-activation effect in the TME, thereby boosting the antitumor efficiency of CAR-NK cells. CK-PSI blocks the TGF-β signaling pathway by Smad3-phosphorylation inhibition and results in reduced ECM deposition in combination with mild photothermal intervention, thus enhancing the infiltration and immune activity of CAR-NK cells synergized by the immune self-activation effect (Figure 3l).

### Cytotoxicity and Immune Activity Detection of CK-PSI *In Vitro*

To evaluate the antitumor immune effect of CK-PSI against NSCLC cells, A549 cells expressing Td-Tomato were selected as target cells and subjected to different treatments, followed by mild NIR-II photothermal treatment in the CK-PSI treated group. After incubation for 24 h, all groups of tumor cells were collected for cytotoxicity detection, and CAR-NK cells were collected for immune activation evaluation (Figure 4a). Tumor cell apoptosis was quantified by flow cytometry analysis. CK-PSI plus laser irradiation treatment exhibited the most apoptotic rate of approximately 70.8% (Figure 4b), which was 4.48-fold and 2.57-fold higher than that of the CAR-NK cell group and CK-PSI group (Figure 4c), respectively. In addition, compared with the CAR-NK + PSI + Laser group, CK-PSI plus laser irradiation treatment exhibited a higher apoptotic rate (Figure S21), ascribed to stronger photothermal effect from PSI NPs by the hitchhiking targeted delivery, indicating the excellent antitumor killing efficiency of CK-PSI biohybrids under NIR-II laser irradiation. To enable visualization of cell apoptosis, A549 cells expressing Td-Tomato were treated with different forms of CAR-NK cells modified by Zs-Green. As shown in Figure 4d, cell clusters of CAR-NK cells and A549 cells were observed in treatment groups, manifesting the targeting affinity of CAR-NK cells toward A549 cells. More importantly, compared with the control group and CAR-NK group, A549 cells suffered from more serious apoptosis in the CK-P + Laser and CK-PSI + Laser groups, accompanied by a drastic reduction of living A549 cells. On the one hand, bioorthogonal conjugation endowed CAR-NK cells with hitchhiking ability for targeting delivery of PSI NPs into tumor cells for boosting immune activity of CAR-NK cell through SIS3 mediated immune regulation (Figure 3l).^43,44^ On the other hand, mild NIR-II PTT further amplified efficiency of immunotherapy through thermal ablation mediated cell destruction.

**Figure 4:**
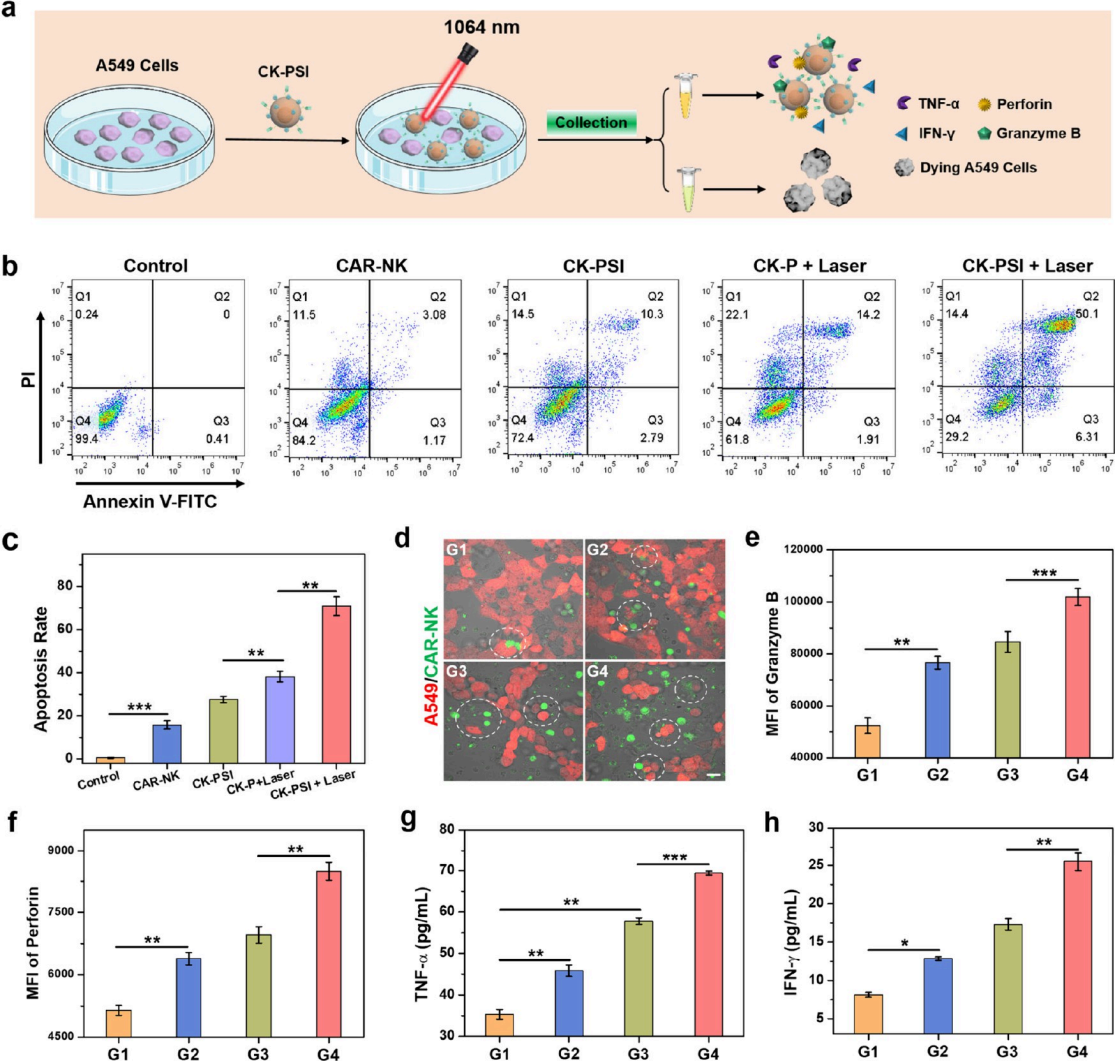
Cytotoxicity analysis and immunoactivity evaluation of CK-PSI. (a) Schematic illustration of CK-PSI mediated immune response and cell killing against A549 cells. (b) Flow cytometric analysis and (c) statistical results of A549 cell apoptosis after various treatments. (d) Fluorescence images of A549 cells and CAR-NK cells with various treatments. Scale bar: 20 μm. Mean fluorescence intensity of (e) granzyme B and (f) perforin secretion after different treatments. Detection of (g) TNF-α and (h) IFN-γ release. G1: CAR-NK, G2: CK-PSI, G3: CK-P + Laser, G4: CK-PSI + Laser. *p < 0.05, **p < 0.01, and ***p < 0.001.

The immune activity of CAR-NK cells was evaluated by degranulation assays (Figure S22), including granzyme B and perforin secretion. Compared with the CAR-NK group, the CK-PSI group showed higher granzyme B and perforin levels (Figure 4e and Figure 4f), indicating that PSI NPs can amplify immune activation of CAR-NK cells after bioorthogonal conjugation. In particular, the granzyme B and perforin levels in the CK-PSI + Laser group were higher than those in the CK-PSI group, possibly ascribed to the elevated SIS3 release triggered by NIR-II photothermal effect. Correspondingly, the CK-PSI plus laser irradiation treated group exhibited the highest cytokine (TNF-α and IFN-γ) release for evoking tumor cell killing compared with other groups (Figure 4g and Figure 4h), verifying that CK-PSI can enhance the antitumor immune activity of CAR-NK cells as well as improved cytokine production under NIR-II laser irradiation. These results confirm that CAR-NK cell biohybrids with hitchhiking NIR-II nanomedicine might be an emerging paradigm for potentiating antitumor therapeutic efficiency of CAR-NK cells by the integration of Smad3 inhibitor and mild NIR-II PTT.

### *In Vivo* Targeting NIR-II FI by CK-PSI Biohybrids

Encouraged by excellent NIR-II FI performance *in vitro*, CK-PSI was injected into mice through the tail vein, and the blood vessels of the mice were immediately lit up. The dynamic distribution of the biohybrid in the systemic vasculature was clearly visualized by NIR-II FI with high imaging resolution (Figure 5a), enabling precise localization tracking of the CAR-NK cells. According to the fluorescence intensity analysis, the diameters of the labeled abdominal and hind femoral vessels were estimated to be approximately 0.628 and 0.711 mm (Figure 5b and Figure 5c), respectively, implying a high signal to background ratio and imaging penetration depth.

**Figure 5:**
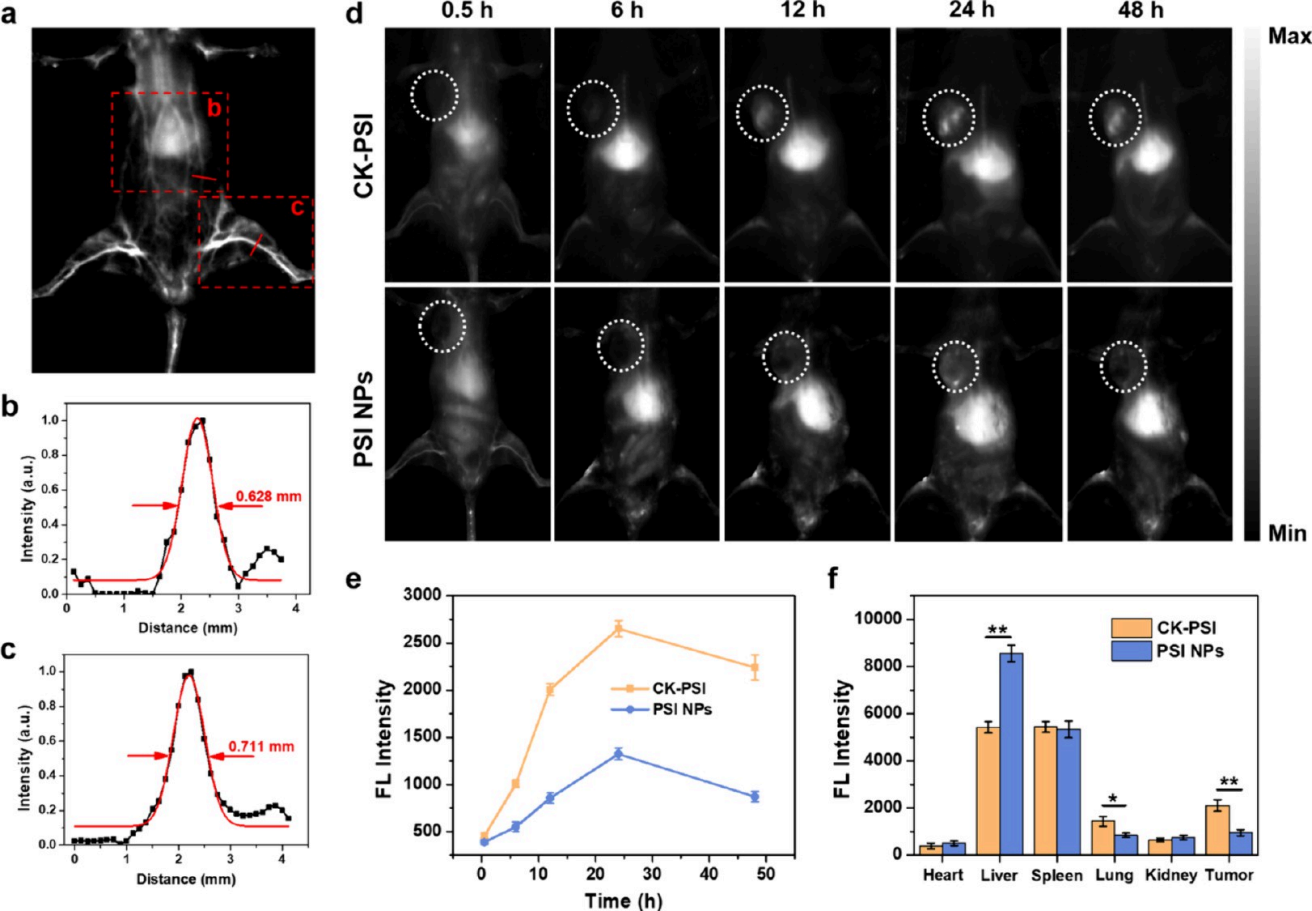
*In vivo* tracking and localization by NIR-II FI. (a) NIR-II FI of mouse blood vessels at 10 min after CK-PSI injection. Fluorescence intensity analysis of labeled (b) abdominal and (c) hind femoral vessels. (d) NIR-II fluorescence images of A549 tumor-bearing mice and (e) tumor fluorescence intensity at indicated time points after intravenous injection with CK-PSI and PSI NPs. (f) NIR-II fluorescence distribution of tumors and major organs at 48 h postinjection. *p < 0.05, **p < 0.01.

To evaluate the *in vivo* targeting NIR-II FI capability, CK-PSI and PSI NPs were intravenously injected into A549 tumor-bearing mice via the tail vein, respectively, and the drug distribution was monitored by time-dependent NIR-II FI. As shown in Figure 5d, the *in vivo* dynamic distribution of CAR-NK cells over time was visualized by NIR-II FI of PSI NPs. CK-PSI exhibited a gradually increased fluorescence signal at the tumor site over time. The tumor tissue could be clearly illuminated at 12 h postinjection and the fluorescence signal reached a peak at 24 h postinjection, which was about 2.0-fold higher than that of the PSI NPs injected group due to the active targeting recognition toward tumor cells provided by CAR-NK cells (Figure 5e). In contrast, the fluorescence signal of the tumor tissue in the PSI NPs-injected group did not significantly fluctuate over time, which relied only on the passive targeting mediated by the enhanced permeability and retention (EPR) effect. At 48 h postinjection, NIR-II FI of tumors and major organs showed the higher fluorescence signal of tumor tissue in the CK-PSI injected group (Figure S23), with an approximately 2.22-fold enhancement compared with PSI NPs injected group (Figure 5f). These results confirm that CK-PSI not only enables the real-time monitoring of CAR-NK cell distribution but also affords precise diagnosis of deep-seated tumors by NIR-II FI.

### *In Vivo* Synergistic Antitumor Immunotherapy

Considering the active targeting ability of CK-PSI toward lung cancer tumors, the immunotherapeutic efficacy of CK-PSI was further evaluated. Immunodeficient NSG mice were subcutaneously inoculated with human A549 cells. After 14 days, four groups of randomly assigned A549 tumor-bearing mice were intravenously injected with PBS, CAR-NK cells, and CK-PSI through the tail vein, respectively (Figure 6a). At 24 h postinjection, the tumors of mice injected with CK-PSI were gently heated with 1064 nm laser irradiation for 10 min under the monitoring of infrared thermal imaging camera (Figure 6b). The tumor temperature rapidly rose to 45 °C within 2 min (Figure 6c), which is favorable for low-temperature PTT as well as TME remodeling, including tumor ECM destruction, vasculature dilation, and hypoxia relief. After various treatments, the tumor volume was measured every 3 days over a period of 15 days, obtaining tumor growth curves (Figure 6d). As shown in Figure 6e, compared to the rapidly increased tumor growth in the PBS group, CAR-NK cell treatment showed a moderate tumor growth inhibition effect by anti-B7H3 CAR mediated targeted immunotherapy. In striking contrast, CK-PSI significantly delayed tumor growth, which was ascribed to the reduced ECM generation and the improved tumor infiltration of CK-PSI by SIS3 mediated TGF-β signaling pathway suppression. In particular, CK-PSI plus laser irradiation treatment showed the highest tumor growth inhibition, which benefited from the TME remodeling mediated by mild NIR-II PTT together with TGF-β signaling blockade. The therapeutic effect of CK-PSI plus laser irradiation was confirmed by the significant difference in the tumor photograph (Figure 6f), with a tumor inhibition rate of up to 86.92% by tumor weight (Figure 6g). After 15 days of treatment, tumor tissues and major organs of mice were collected for pathological analysis. H&E and TUNEL staining showed more severe tumor cell damage and necrosis in the CK-PSI NPs group and the CK-PSI + Laser group (Figure 6i and Figure 6j). The negligible pathological differences for major organs (heart, liver, spleen, lung, and kidney) were observed between the PBS group and the CK-PSI + Laser group by H&E staining after 15 days (Figure S24). The levels of blood biochemical markers, including alanine aminotransferase (ALT), aspartate aminotransferase (AST), creatinine (CREA), and UREA showed almost no differences compared to the PBS group after various treatments (Figure S25). Stable body weight was observed in all groups throughout the treatment period (Figure 6h), manifesting the biocompatibility and safety of CK-PSI plus NIR-II laser irradiation. These results imply that PSI NPs backpacks equipped on the surface of CAR-NK cells enable improved tumor infiltration and boosted immune response of CAR-NK cells through mild NIR-II photothermal intervention and TGF-β signaling blockade, thereby strengthening the efficiency of CAR-NK cell therapy against lung tumors.

**Figure 6:**
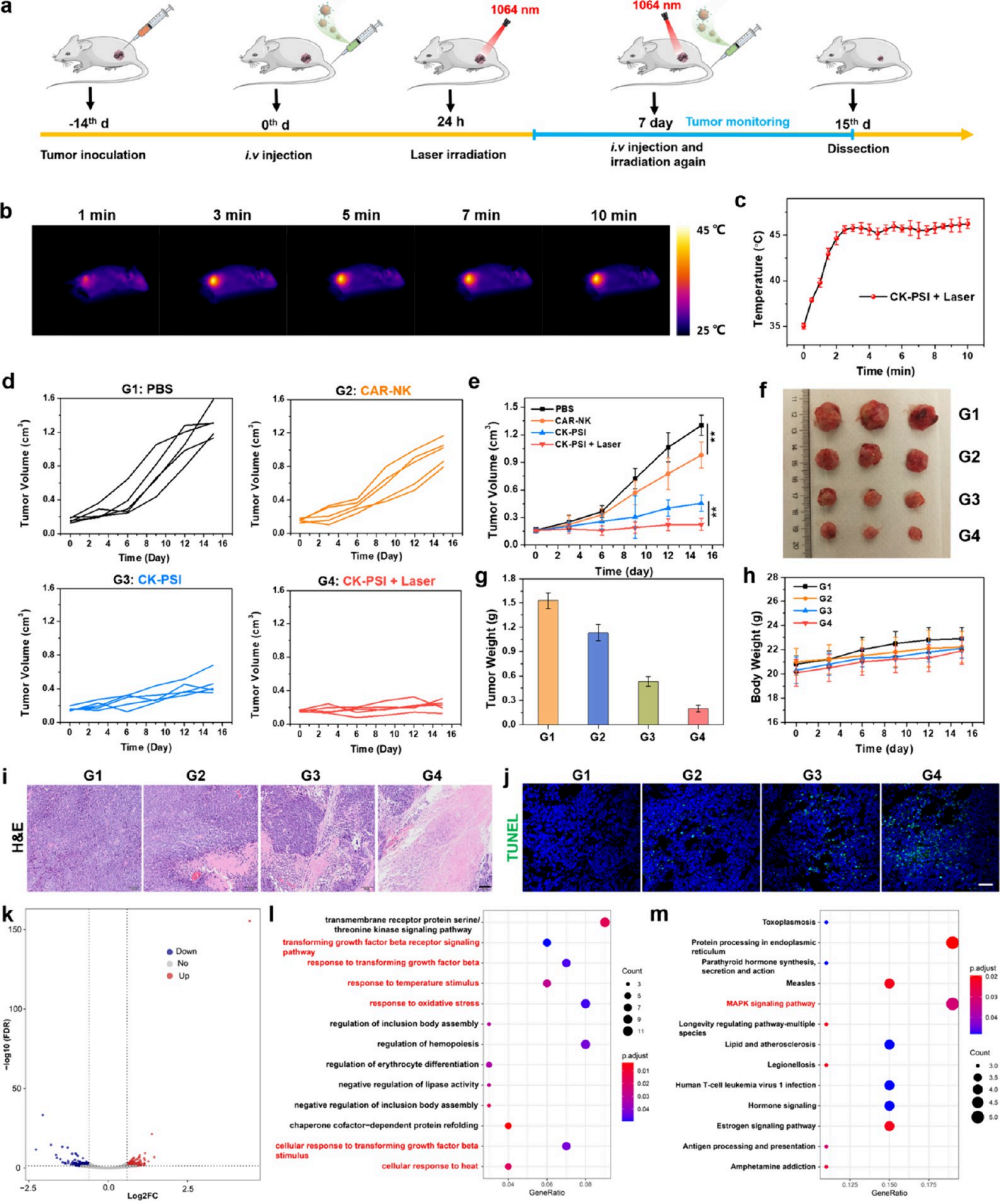
*In vivo* synergistic antitumor immunotherapy. (a) Description of the therapeutic protocol. (b) Photothermal images of tumors in the group injected with CK-PSI under 1064 nm laser irradiation. (c) Temperature rise curves corresponding to (b). (d, e) Tumor growth curves of different treatment groups. (f) The tumor photograph and (g) tumor weight after 15 days of treatment. (h) Mouse body weight during various treatments. (i) H&E staining images (scale bar: 100 μm) and (j) TUNEL staining images (scale bar: 50 μm) of tumors from different treatment groups. (k) Volcano map of DEGs between the PBS group and the CK-PSI + Laser group. (l) Bubble diagram of GO enrichment analysis of DEGs. (m) Bubble diagram of KEGG enrichment analysis of the upregulated DEGs after treatment with CK-PSI + Laser. G1: PBS, G2: CAR-NK, G3: CK-PSI, G4: CK-PSI + Laser. **p < 0.01.

### Potential Therapeutic Mechanisms of Synergistic Immunotherapy

To reveal the potential molecular mechanisms of CK-PSI mediated amplified immunotherapy, mRNA sequencing of tumor tissues from the PBS group and the CK-PSI + Laser group was performed. All genetic data were first normalized in the DESeq2 analysis platform and then further analyzed (Figure S26). Differentially expressed genes (DEGs) were identified after CK-PSI + Laser treatment (Figure 6k and Figure S27a). Gene ontology (GO) enrichment analysis showed that CK-PSI + Laser treatment significantly altered the genes associated with response to TGF-β and response to heat within tumor tissues, accompanied by significant changes in genes of the TGF-β signaling pathway (Figure 6l). This may be attributed to SIS3 hitchhiking on CK-PSI mediated inhibition of Smad3-phosphorylation, thereby affecting the response of CAFs to TGF-β. Mild photothermal treatment induced by NIR-II laser irradiation could evoke the thermal response of tumor cells, evidenced by the changes of genes associated with cellular response to heat. The upregulation of genes associated with the vascular endothelial growth factor receptor signaling pathway indicated that TGF-β signaling blockade and mild NIR-II photothermal intervention synergistically reduced CAFs activation and ECM deposition, promoting vascular dilation and intratumoral infiltration of CAR-NK biohybrids (Figure S28). Enhanced CAR-NK cell infiltration and immune activation significantly upregulated genes related to the cytokine-mediated signaling pathway and tumor necrosis factor-mediated signaling pathway, triggering strong oxidative stress and inducing tumor apoptosis. Kyoto Encyclopedia of Genes and Genomes (KEGG) pathway enrichment analysis showed the activation of MAPK signaling pathways related to the growth, differentiation, and survival of tumor cells (Figure 6m), demonstrating the synergistic antitumor therapeutic effect of CK-PSI plus NIR-II laser irradiation. Protein–protein interaction (PPI) network analysis indicated that heat-responsive proteins (such as HSPB1, HSPA1B, and HSPH1) and collagens (such as COL1A2, COL12A1, and COL3A1) play an important role in CK-PSI + Laser mediated TME remodeling, including CAFs remodeling and ECM reduction (Figure S27b). Taken together, the results presented above demonstrate that CK-PSI can effectively promote the infiltration and immune activity of CAR-NK cells through TGF-β signaling inhibition and mild NIR-II photothermal intervention, thereby potentiating the efficiency of immunotherapy.

### *In Vivo* TME Remodeling and Amplified Immune Activation Effect Evaluation

The therapeutic effect of CAR-NK cells toward solid tumors is hindered by the immunosuppressive TME, including TGF-β signaling induced CAFs activation and ECM deposition with compressed vasculature and severe hypoxia. Mild NIR-II photothermal intervention and TGF-β signaling blockade can promote the intratumoral infiltration and immune activation of CK-PSI through TME reconstruction (Figure 7a). In order to explore how CK-PSI regulated TME to potentiate the efficiency of CAR-NK cell immunotherapy, the levels of various TME-related components within lung cancer tumors were evaluated after various treatments. The tumors treated with CK-PSI with or without laser irradiation exhibited significantly reduced fluorescence signals of α-SMA compared with the PBS group and CAR-NK group (Figure 7b and Figure 7c), holding direct evidence of low activation of CAFs due to PSI NPs mediated Smad3-phosphorylation inhibition. As a basic ingredient of the ECM, the expression of collagen I was detected after various treatments. High cross-linking of tumor cells and collagen fibers was observed in the PBS group and the CAR-NK group (Figure 7b), which hinders the diffusion of CAR-NK cells into the tumor tissue, leading to insufficient immune response. However, the expression of collagen I was significantly reduced in the CK-PSI group and the CK-PSI + Laser group, obtaining inhibition rates of 50.79% and 84.93%, respectively, compared with the PBS group (Figure 7d). This demonstrates that mild NIR-II photothermal intervention and TGF-β signaling obstruction can break the physical barrier of tumor tissues by destroying the ECM, providing a favorable TME for the infiltration and penetration of CK-PSI. In addition, remarkably obvious vascular dilation accompanied by increased lumen diameter was observed in the CK-PSI + Laser group, which was verified by the increased expression of vascular endothelial marker CD31 (Figure 7e), with an approximately 5.36-fold enhancement of fluorescence intensity compared with the PBS group (Figure 7g). The ECM destruction and tumor vessel dilation can not only facilitate more oxygen delivery to relieve tumor hypoxia but also enable improved infiltration of CK-PSI into solid tumors through the elimination of physical obstacles. A weak hypoxia inducible factor-1α (HIF-1α) fluorescence signal was observed in the CK-PSI treated group (Figure 7f), which may be ascribed to ECM reduction induced by TGF-β signaling inhibition.^23^ It was worth noting that mild NIR-II photothermal treatment in the CK-PSI + Laser group further provoked significant hypoxia relief through the weakest fluorescence signal of HIF-1α, which decreased by approximately 5.96-fold compared with the PBS group (Figure 7h). These results verify that CK-PSI remodels immunosuppressive TME to create a favorable soil for enhancing the immune response of CAR-NK cells through the synergistic effect of mild NIR-II photothermal treatment and TGF-β signaling inhibition. CAR-NK cell mediated antitumor immune activity was investigated by degranulation and cytokine release assays. The CK-PSI + Laser group showed the highest secretion of granzyme B and perforin by the brightest fluorescence signals (Figure 7i). Moreover, CK-PSI plus laser irradiation resulted in the highest release of cytokines TNF-α and IFN-γ (Figure 7j and Figure 7k), indicating amplified antitumor immune response.

**Figure 7:**
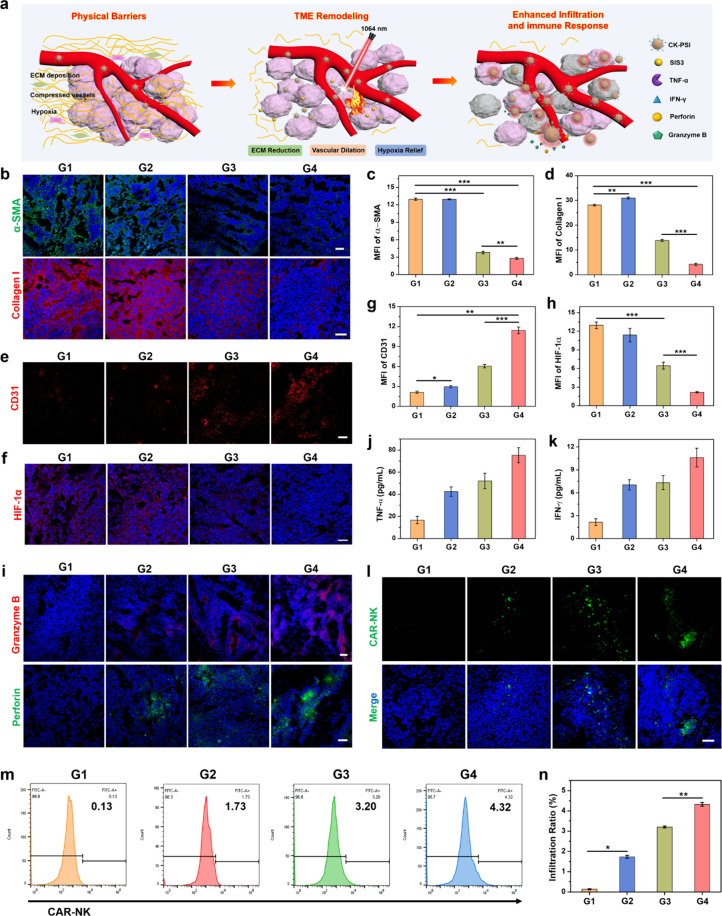
TME remodeling mediated amplified immune response. (a) Schematic illustration of TME remodeling mediated enhanced infiltration and immune activation of CK-PSI against solid tumors. (b) Immunofluorescence images of α-SMA and collagen I expression and (c, d) corresponding fluorescence intensity analysis within tumor tissues after different treatments. Scale bar: 50 μm. Immunofluorescence images of (e) tumor vasculature and (f) HIF-1α, and (g, h) fluorescence quantification. Scale bar: 50 μm. (i) Immunofluorescence images of granzyme B and perforin secretion. Scale bar: 50 μm. Detection of (j) TNF-α and (k) IFN-γ release in mouse serum after various treatments. (l) Fluorescence images of CAR-NK cell infiltration in tumors. Scale bar: 50 μm. (m) Flow cytometric analysis and (n) statistical data of intratumoral infiltration of CAR-NK cells after various treatments. G1: PBS, G2: CAR-NK, G3: CK-PSI, G4: CK-PSI + Laser. *p < 0.05, **p < 0.01, and ***p < 0.001.

In order to investigate the intratumoral infiltration effect of CAR-NK cells, A549 tumor-bearing mice were intravenously injected with CAR-NK cells and CK-PSI with fluorescence protein Zs-Green, followed by irradiation with a 1064 nm laser for 10 min. As shown in Figure 7l, the CK-PSI group and the CK-PSI + Laser group exhibited more CAR-NK cell infiltration in tumor tissues by the fluorescence images of CAR-NK cells. Flow cytometry analysis revealed that the CK-PSI plus laser irradiation treated group retained the most CAR-NK cells within tumors compared with other groups after 15 days of treatment (Figure 7m). The retention and infiltration ratios of CAR-NK cells were approximately 2.50-fold and 1.35-fold higher than those of the CAR-NK group and CK-PSI group, respectively (Figure 7n). The above results verify that CK-PSI can promote the intratumoral infiltration of CAR-NK cells and boost antitumor immunofeedback effects by reshaping the immunosuppressive TME under NIR-II laser irradiation.

### *In Vivo* Antitumor Therapy in Humanized Mouse Models

Given the excellent therapeutic effect of CK-PSI on immunodeficient A549 tumor-bearing mice, we further investigated its antitumor therapeutic potential toward humanized mouse models. Peripheral blood mononuclear cells (PBMCs) were injected into NSG mice with immunodeficiency to rebuild their immune system. After about 2 weeks, the percentage of humanized immune cells in the peripheral blood of the mice reached about 35.2% (Figure S29), indicating the successful establishment of humanized mouse models. A549 tumor-bearing humanized mice underwent different treatments. At 24 h postinjection, the tumors in PSI NPs and CK-PSI-injected groups were mildly heated by 1064 nm laser irradiation for 10 min. Tumor temperature was monitored by infrared thermal images, and the temperature slowly rose to 45 °C and remained stable (Figure S30). A549 tumor-bearing humanized mice underwent different treatments. As shown in Figure S31a and Figure S31b, tumor growth curves were obtained by recording tumor volumes every 3 days after various treatments. CAR-NK cells and PSI NPs + Laser treatments exhibited certain tumor growth inhibition, attributed to cell immunotherapy and mild NIR-II PTT, respectively. More significant growth inhibition was observed in the CK-PSI treated group, with a tumor inhibition rate of approximately 69.11% through tumor weight (Figure S31c), due to improved infiltration and antitumor killing efficiency of CAR-NK cells via TME remodeling. In particular, the mild photothermal effect induced by NIR-II laser irradiation further enhanced the antitumor effect of CK-PSI, with the highest tumor growth inhibition. During 15 days of treatment, mouse body weight remained stable in all groups without significant differences (Figure S31d), indicating the biocompatibility of various treatments. These results confirm the outstanding antitumor therapeutic effect of CK-PSI in humanized mouse models and great potential for clinical application.

## Conclusion

In summary, we designed and synthesized NIR-II semiconducting polymers with excellent optical performance through donor doping adjustment, followed by use for the fabrication of NIR-II nanomedicine (PSI NPs) together with the Smad3 inhibitor (SIS3) with immunomodulatory capabilities. Nanoengineered CAR-NK biohybrids (CK-PSI) were prepared through bioorthogonal reactions. CK-PSI served as a living cell carrier to enable targeted delivery of NIR-II nanomedicine into solid tumors, which provided real-time monitoring of CAR-NK cell distribution and precise localization of deep-seated tumors via NIR-II FI. In addition, CK-PSI effectively overcame physical barriers of solid tumors through mild NIR-II photothermal intervention and TGF-β signaling inhibition. TME remodeling promoted the intratumoral infiltration and proliferation of CAR-NK cells, thereby boosting the antitumor immune response of CAR-NK cells. The nanoengineered living cell theranostic platform with the integration of NIR-II phototheranostics and TME remodeling paves a new way for promoting targeted drug delivery and potentiating efficiency of cell-based immunotherapy against solid tumors.

## Supplementary Material



## Notes

Detailed experimental procedures; ^1^H NMR of polymers; DFT calculations; absorption and fluorescence emission spectra; zeta potential; photothermal images; cell fluorescence images; flow cytometry analysis; NIR-II fluorescence images; H&E staining images; mRNA sequencing analysis; antitumor therapy (PDF)
