# Supplementary material for: NIR-II Nanomedicine Engineered CAR-NK Cells for Precision Navigation and Potentiating Lung Cancer Immunotherapy by Remodeling Tumor Microenvironment
Source: J Am Chem Soc. 2026 Mar 6;148(10):11317–32. doi: 10.1021/jacs.6c01128 (PMC13003482; doi:10.1021/jacs.6c01128)
Supplement: Supplementary file 1 [file ja6c01128_si_001.pdf]

NIR-II nanomedicine engineered CAR-NK cells for precision navigation and potentiating lung cancer immunotherapy by remodeling tumor microenvironment

Yeneng Dai, [a,b] *Qihang Ding,* [d,*] *Ze Chen,* [a] *Guanda Jiao,* [a] *Yiqi Yang,* [a] *Shengyu Fu,* [a] *Ziyi Yang,* [a] *Xiaoxi Liu,* [a] *Kun Qian,* [c] *Zhen Cheng,* [c,*] *Dongliang Leng,* [a,*] *Qi Zhao* [a,b,*]

[a] Cancer Centre, Institute of Translational Medicine, Faculty of Health Sciences, University of Macau, Macau SAR 999078, China.

[b] MoE Frontiers Science Center for Precision Oncology, University of Macau, Taipa, Macau SAR 999078, China.

[c] State Key Laboratory of Drug Research, Molecular Imaging Center, Shanghai Institute of Materia Medica, Chinese Academy of Sciences, Shanghai 201203, China. Shandong Laboratory of Yantai Drug Discovery, Bohai Rim Advanced Research Institute for Drug Discovery, Yantai, Shandong 264117, China.

[d] Department of Chemistry, Korea University, Seoul 02841, Korea.

Experimental section

Materials. Cholesterol, 1,2-dipalmitoyl-sn-glycero-3-phosphocholine (DPPC), 1, 2-distearoylsn-glycero-3-phosphoethanolamine-N-[dibenzocyclooctyl (polyethylene glycol)-2000] (DSPE-PEG_{2000}-DBCO) were purchased from Shanghai Ponsure Biotech, Inc. Azide sugar ($Ac_4GalNAz$) were obtained from Click Chemistry Tools (Scottsdale, AZ, USA). 6,7-Bis(4-(hexyloxy)phenyl)-4,9-di(thiophen-2-yl)- [1,2,5]thiadiazolo [3,4-g]quinoxaline (TTQ), (4,4'-di-n-dodecyl-2,2'-bithiophene-5,5'-diyl)bis(trimethylstannane) (TC) and 2,5-bis(trimethylstannyl) thieno [3,2-b]thiophene (DT) were purchased from SunaTech Inc. SIS3 was purchased from Ambeed. Cell Counting Kit-8 (CCK-8) and Annexin V-FITC/PI Cell Apoptosis Detection Kit were obtained from Yeasen Biotechnology CO., Ltd. PE-anti-granzyme B, PE/Cy7-anti-perforin and APC-anti-CD56 were purchased from BioLegend. TNF-α and IFN-γ ELISA Kit was bought from Beijing Solarbio Science & Technology Co., Ltd.

Characterization. The ^{1}H NMR data were obtained utilizing a Bruker Ultra Shield Plus NMR instrument (400 MHz). Gel permeation chromatography (GPC) was used to determine the molecular weight, polydispersity (PDI) and polymerization degree of the polymer. The morphology of PSI NPs and CK-PSI were determined by a transmission electron microscope (HT7700, TEM) and a scanning electron microscope (5000X+UltimMax 40e), respectively. The size and Zeta potential of PSI NPs were examined by Zetasizer Nano ZS (Malvern, UK). UV-vis absorption spectra and NIR-II fluorescence spectra were determined using a PerkinElmer Lambda UV spectrophotometer and a NIR-II spectrophotometer (Fluorolog 3, Horiba). NIR-II Fluorescence imaging was performed and analyzed by AniView Phoenix Full Spectrum Animal *In Vivo* Imaging System (Guangzhou Biolight Biotechnology Co., Ltd.).

Etiological analysis of fibrotic lung cancer. We compared the expression difference of extracellular matrix (ECM) between lung cancer and normal lung tissues by bioinformatics analysis using the TCGA database. To investigate the relationship between cancer-associated fibroblasts (CAFs) activation and ECM deposition, we analyzed the correlation between ACTA2 gene and fibronectin or collagen I expression in TCGA lung cancer. In addition, to verify the mechanism of lung cancer fibrosis, human embryonic lung fibroblasts cell line MRC-5 cells were cultured with conditioned medium from lung cancer A549 cells to generate CAFs, and the expression of Smad3-phosphorylation, α-SMA and collagen I in CAFs were detected by western blot analysis and

immunofluorescence staining.

Generation and azide labeling of anti-B7H3 CAR-NK cells. CAR-NK cells were generated according to our previously works.[1, 2] Briefly, the CAR containing anti-B7-H3 single chain variable region (scFv), the CD8 transmembrane (TM) region and the intracellular domains of 4-1BB and CD3ζ, were ligated to a lentiviral expression vector with Zs-Green. The generated anti-B7H3 scFv plasmid was used to transfect HEK293T cells together with packaging plasmids, psPAX2 and pMD2.G, producing the lentiviral particles. NK-92 cells were transfected with the concentrated lentiviral particles, followed by further expanded and sorted with a FACSAria II cell sorter (BD Biosciences), obtaining CAR-expressed NK-92 cells. To fabricate azide (N_3)-labeled CAR-NK cells, 50 μM $Ac_4GalNAz$ was added to the culture medium containing CAR-NK cells, followed by incubation for 3 days, obtaining CAR-NK-N_3.

Synthesis of NIR-II semiconducting polymers. NIR-II semiconducting polymers (SPs) were synthesized via Stille conjugation polymerization. For the synthesis of SP1, the donor 2,5-bis(trimethylstannyl) thieno [3,2-b]thiophene (DT) (5.399 mg, 0.0116 mmol), (4,4'-di-n-dodecyl-2,2'-bithiophene-5,5'-diyl)bis(trimethylstannane) (TC) (4.802 mg, 0.0058 mmol) and the acceptor 6,7-Bis(4-(hexyloxy)phenyl)-4,9-di(thiophen-2-yl)- [1,2,5]thiadiazolo [3,4-g]quinoxaline (TTQ) (15 mg, 0.0174 mmol) were added to 10 mL anhydrous toluene in a 100 mL three-necked flask. The flask was degassed and recharged with nitrogen three times after adding catalysts bis(triphenylphosphine)palladium(II) dichloride (8 mg, 0.011 mmol) and 2,6-di-tertbutylphenol (5 mg, 0.024 mmol). The polymerization was performed at 100 °C in the dark under nitrogen atmosphere, accompanied by constant stirring. After 1 h, the mixed solution was cooled to room temperature, followed by added with excess methanol to produce precipitation. After further filtration and drying, the polymer SP1 was obtained.

The other two semiconducting polymers were synthesized in the same way as SP1 by adjusting the ratio of donor and acceptor. For SP2, DT (4.049 mg, 0.00869 mmol), TC (7.202 mg, 0.00869 mmol) and TTQ (15 mg, 0.0174 mmol) were used for polymerization. For SP3, DT (2.70 mg, 0.0058 mmol), TC (9.603 mg, 0.0116 mmol) and TTQ (15 mg, 0.0174 mmol) were used for polymerization.

Preparation of DBCO-modified NIR-II liposomal nanomedicine (PSI NPs). To prepare NIR-II liposomal nanomedicine with DBCO modification, 10 mg DSPE-PEG_{2000}-DBCO, 10 mg DPPC and 3 mg cholesterol were dissolved in 10 mL chloroform in a round-bottom flask. Then, 500 μL

polymer SP3 (1 mg/mL, in THF) and 200 μL Smad3 inhibitor SIS3 (1 mg/mL, in chloroform) were added to the lipid solution. After thorough mixing, the organic solvent was removed by rotation and evaporation for 0.5 h at 30 rpm at 40 °C using a rotary evaporator, forming a lipid film. The lipid film was hydrated under continuous ultrasound by adding 4 mL ultrapure water. After further ultrafiltration (10 KDa, 4500 rpm) three times, polymer-SIS3 lipid nanomedicine (PSI NPs) with DBCO modification were obtained. In order to acquire nanoparticles with uniform size, the PSI NPs was extruded through a 0.22 μm polycarbonate membrane.

Preparation and characterization of CK-PSI. PSI NPs were conjugated onto the surface of CAR-NK cells via a bioorthogonal reaction. Briefly, PSI NPs with different concentrations of DSPE-PEG_{2000}-DBCO were added to the culture medium of CAR-NK-N_3 cells (1×10^6 cells), followed by incubation for 1 h. After washed and centrifuged 3 times with PBS to remove unbound PSI NPs, PSI NPs-conjugated CAR-NK cells (CK-PSI) were obtained. CK-P was obtained by encapsulating the polymer SP3 into liposomes, followed by conjugated to CAR-NK cells.

To investigate the conjugation efficiency of liposomes to CAR-NK cells, DiR encapsulated PSI NPs containing different concentrations of DSPE-PEG_{2000}-DBCO were incubated with CAR-NK-N_3 (1×10^6 cells) for 1 h, and the conjugation efficiency was analyzed by flow cytometry. To investigate the binding affinity of CAR-NK cells and CK-PSI against B7H3 antigen, His-tagged B7H3 was incubated with CAR-NK cells and CK-PSI (1×10^6 cells) for 1 h, respectively. After washed with PBS, the cells were incubated with fluorescence labeled anti-His antibody for 1 h. The binding affinity was assessed by flow cytometry.

NIR-II photothermal effect and photothermal-induced SIS3 release of PSI NPs. To evaluate the photothermal effect of PSI NPs, PSI NPs aqueous solutions with different polymer concentrations were exposed to 1064 nm laser irradiation (1 W/cm^2) for 5 min. The temperature changes of the solutions were recorded using an infrared thermal imaging camera. For photothermal stability evaluation, the PSI NPs solutions were cooled to room temperature after the laser was turned off, and the temperature changes were monitored during continuous laser on/off cycles. In addition, laser irradiation with different power intensity was implemented to evaluate the power-dependent photothermal effect. In order to investigate the photothermal-induced release behavior of SIS3 from thermosensitive liposomes, the PSI NPs solution was exposed to 1064 nm laser irradiation through continuous on-off cycles (1 W/cm^2, 5 min). Drug release was monitored by

absorption of SIS3.

Thermal tolerance and cell viability assay of CK-PSI. To investigate the influence of PSI NPs conjugation on the viability of CAR-NK cells, PSI NPs containing different concentrations (25, 50, 100, 200, 300 μg/mL) were conjugated with CAR-NK cells, and the cell viability of CK-PSI was detected by cell counting kit-8 (CCK-8) assay, and the cytokines (TNF-α and IFN-γ) released from CAR-NK cells were detected using enzyme linked immunosorbent assay (ELISA) kit. To evaluate the heat resistance, CK-PSI and tumor cells (A549 cells) were incubated for 10 min at different temperatures by water bath heating and under 1064 nm laser irradiation, and the cell viability was quantified using CCK-8 assay.

Intracellular TGF-β signaling inhibition of CAFs. CAFs were first established by culturing MRC-5 cells with conditioned medium from lung cancer A549 cells, followed by treatment with CAR-NK cells, PSI NPs, CK-PSI (SIS3: 3 μg/mL) with or without 1064 nm laser irradiation (1 W/cm^2) for 5 min. Then, CAFs were washed three times with PBS and fixed with 4% paraformaldehyde for 25 min, followed by permeabilized with 0.25% Triton X-100 for 25 min. After blocked with 2.5% bovine serum albumin (BSA) for 2 h and washed three times with PBS, the cells were incubated with α-SMA primary antibody (BF9212, Affinity) and collagen I primary antibody (AF7001, Affinity) overnight at 4 °C, and then the cells were labeled with corresponding secondary antibody with fluorescence for 2 h at room temperature. After staining the nuclei with Hoechst 33342, intracellular α-SMA and collagen I was visualized using CLSM.

After the same treatments as above, the CAFs in all groups were lysed with radioimmunoprecipitation assay (RIPA) lysis buffer and centrifuged at 12,000 × g for 5 min. The supernatant was collected and proteins were separated by 10% SDS-PAGE, followed by transferred to the polyvinylidene fluoride (PVDF) membrane. The 5% skim milk powder was used to block the membrane, followed by incubation with p-Smad3 primary antibody (AF3362, Affinity) or Smad3 primary antibody (AF6362, Affinity), and then further incubated with HRP goat anti-rabbit IgG (H+L). The protein bands are detected using an enhanced chemiluminescence system.

Targeted penetration of CK-PSI. According to the previously reported literature,[3] ECM-enriched 3D tumor spheroid models were established to evaluate the targeted penetration effect of CK-PSI within solid tumors. A549 cells carrying the reporter gene Td-Tomato were mixed with MRC-5 cells at a ratio of 2:1 and seeded to a sphere-ultra-low adsorption surface 96-well plate (Thermo

Fisher Scientific, USA), followed by centrifuged at 300 *g* for 5 min. After 7 days of incubation in a humidified incubator at 37 °C and 5% CO_2, the formed 3D multicellular spheroids were washed with PBS, followed by the addition of NK cells, CAR-NK cells and CK-PSI labeled with Zs-Green for further incubation for 48 h. CK-PSI treated cell spheroids were gently heated under 1064 nm laser irradiation (1 W/cm^2) for 5 min. After washed with PBS three times, the infiltration of CK-PSI in 3D tumor spheroids was observed by fluorescence imaging using laser confocal scanning microscopy (CLSM), and the fluorescence intensity was further quantified at different cell depths.

Immune activation of CAR-NK cells mediated by PSI NPs. To investigate the reversal of the immune activity of CAR-NK cells by PSI NPs under TGF-β stimulation. CAR-NK cells (5 × 10^5 cells/well) were first incubated with TGF-β1 (5 ng/mL) for 6 h, followed by the addition of SIS3 and PSI NPs (SIS3: 10 μg/mL) for further incubation for 24 h. The cell supernatant was collected for the detection of IFN-γ levels. The expression of activating receptor NKp46 on the surface of CAR-NK cells was detected by flow cytometry analysis.

Cytotoxicity and degranulation analysis of CK-PSI. A549 cells labeled with Td-Tomato were seeded in confocal dishes and incubated for 24 h, followed by the addition of CAR-NK cells, CK-P and CK-PSI for further incubation for 24 h. All CAR-NK cells were labeled with Zs-Green protein. CK-PSI treated cells were gently heated by 1064 nm laser irradiation (1 W/cm^2) for 5 min. The recognition and killing effects of CAR-NK cells towards A549 cell were observed by CLSM. In addition, A549 cells without fluorescent labeling were subjected to the same treatment as above. The apoptosis of A549 cells was quantitatively analyzed by flow cytometry using an Annexin V-FITC/PI Cell Apoptosis Detection Kit. To investigate the degranulation and cytokine release of effector cells. After various treatments, the cell suspension was collected for cytokine release analysis (TNF-α and IFN-γ) using an ELISA kit. Effector cells were collected, fixed and permeabilized, followed by staining with PE-anti-granzyme B (Biolegend), PE/Cy7-anti-perforin (Biolegend) and APC-anti-CD56 (Biolegend) for the determination of degranulation by flow cytometry.

Animal Models. NSG mice were provided by Animal Research Core of Faculty of Health Sciences, University of Macau. All animal experiments were approved by the Institutional Animal Care and Use Committee of Macau University, and performed in accordance with the approved protocol (UMARE-041-2020) by the University of Macau Animal Ethics Committee.

***In vitro* and *in vivo* NIR-II FI.** NIR-II FI of free polymer, PSI NPs aqueous solution and CK-PSI cell solutions with different concentrations was detected by AniView Phoenix Full Spectrum Animal *In Vivo* Imaging System (Guangzhou Biolight Biotechnology Co., Ltd.). The dynamic distribution of CK-PSI in mouse blood vessels was detected by NIR-II FI, and the vessel diameter was estimated by Image J software.

To further evaluate the hitchhiking function provided by CAR-NK cells for targeted delivery of PSI NPs, A549 tumor-bearing NSG mice were intravenously injected with PSI NPs and CK-PSI NPs (1 mg/mL, 200 μL) via the tail vein, respectively, followed by imaged under 808 nm laser excitation at different time points. The major organs and tumors of mice were collected for NIR-II FI at 48 h post-injection. All images were acquired through a 1075 nm long pass (LP) filter, and the fluorescence intensity of tumors and main organs was analyzed by analysis software in the imaging system.

***In vivo* anti-tumor immunotherapy.** To evaluate the anti-tumor therapeutic effect of CK-PSI, A549 tumor-bearing NSG mice with immunodeficiency were randomly divided into four groups and treated with PBS, CAR-NK, CK-PSI, and CK-PSI + Laser, respectively. All samples were intravenously injected into mice via the tail vein (CAR-NK cells: 1×10^7 cells/mouse), and the tumors of the CK-PSI injected group were subjected to mild photothermal heating by 1064 nm laser irradiation (1 W/cm^2) for 10 min at 24 h post-injection, and photothermal images were obtained by an infrared thermal imaging camera. After 7 days of treatment, the above treatments was repeated once. The tumor volume and the body weight of the mice were recorded every 3 days during the whole treatment period. After 15 days of treatment, the tumors of the mice were photographed and weighed, followed by hematoxylin-eosin (H&E) staining and the terminal deoxynucleotidyl transferase dUTP nick end labeling (TUNEL) staining together with the main organs.

***In vivo* TME remodeling and immune activation.** To evaluate the tumor microenvironment remodeling efficiency mediated by mild NIR-II photothermal effect and TGF-β signaling inhibition, all groups of tumors were collected for the evaluation of α-SMA, collagen I, HIF-α and CD31 expression after 15 days of treatment. Briefly, the fixed tumor sections were permeabilized and blocked with 0.25% Triton X-100 and 5% BSA, respectively, and then incubated with primary antibodies of α-SMA, collagen I, HIF-1α and CD31 overnight at 4°C. After labeled with corresponding fluorescent secondary antibodies and nuclear staining with Hoechst 33342, tumor

sections were observed under CLSM.

To evaluate infiltration and activation effect of CAR-NK cell, after 15 days of treatment, the tumors were collected and prepared into single-cell suspensions for CAR-NK infiltration detection by flow cytometry. The infiltration of CAR-NK cell in solid tumors was further visualized by fluorescence images of tumor sections under CLSM. In addition, tumor sections were stained with PE-anti-granzyme B (Biolegend) and PE/Cy7-anti-perforin (Biolegend) overnight for degranulation evaluation through the immunofluorescence staining. After 15 days of various treatments, mouse sera were collected for cytokine (TNF-α and IFN-γ) detection using ELISA kits.

***In vivo* anti-tumor therapy against humanized mouse models.** To establish a humanized mouse model, the peripheral blood mononuclear cells (PBMC) were injected into NSG mice (1×10^7 cells/mouse). At 2 weeks post-injection, humanized immune cells in the peripheral blood of the mice were analyzed. After the humanized mouse model was established, A549 cells were inoculated into mice and mice were randomly divided into 5 groups to undergo the following treatments: (1) PBS, (2) CAR-NK, (3) CK-PSI, (4) PSI NPs + Laser, (5) CK-PSI + Laser. All samples were intravenously injected into mice via the tail vein (CAR-NK cells: 1×10^7 cells/mouse). Tumors in the PSI NPs and CK-PSI groups were gently heated by 1064 nm laser irradiation (1 W/cm^2) for 10 min at 24 h post-injection. After 7 days of treatment, the above treatments were repeated once. Tumor volume and mouse weight were monitored every 3 days. After 15 days of treatment, tumors in all groups were collected and weighed to evaluate the therapeutic efficiency.

Statistical analysis. The experiments were repeated at least three times. All results were obtained as means and standard deviations calculated from the measurements by repeated experiments. Data were analyzed via ANOVA and statistical significance was provided. (*$p < 0.05$, **$p < 0.01$ and ***$p < 0.001$)

Supplementary Figures

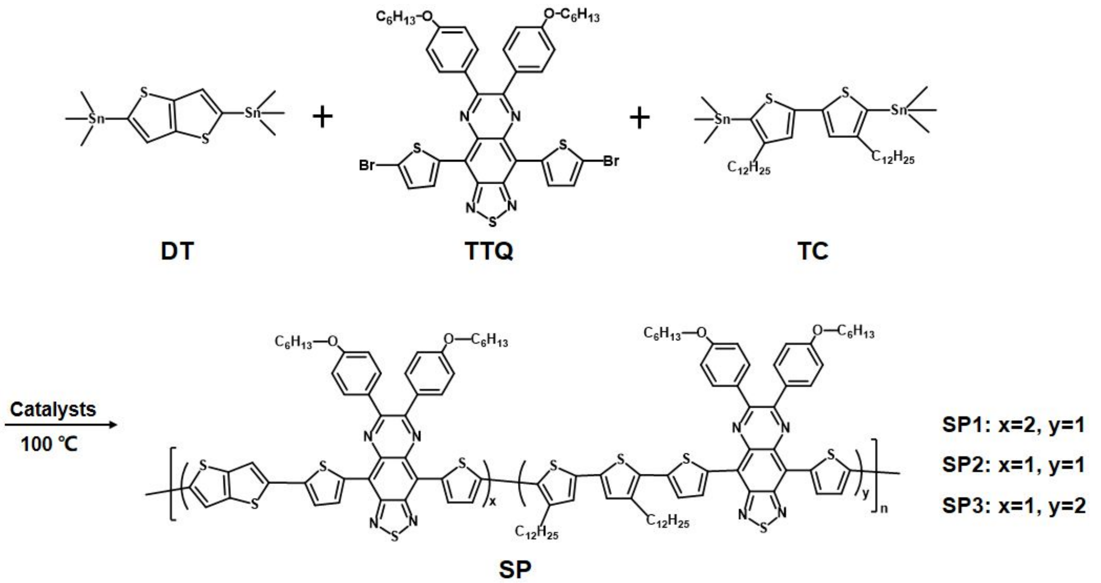

Scheme S1. Synthesis route of NIR-II semiconducting polymers. Catalysts: Bis(triphenylphosphine)palladium(II) dichloride and 2,6-Di-tertbutylphenol.

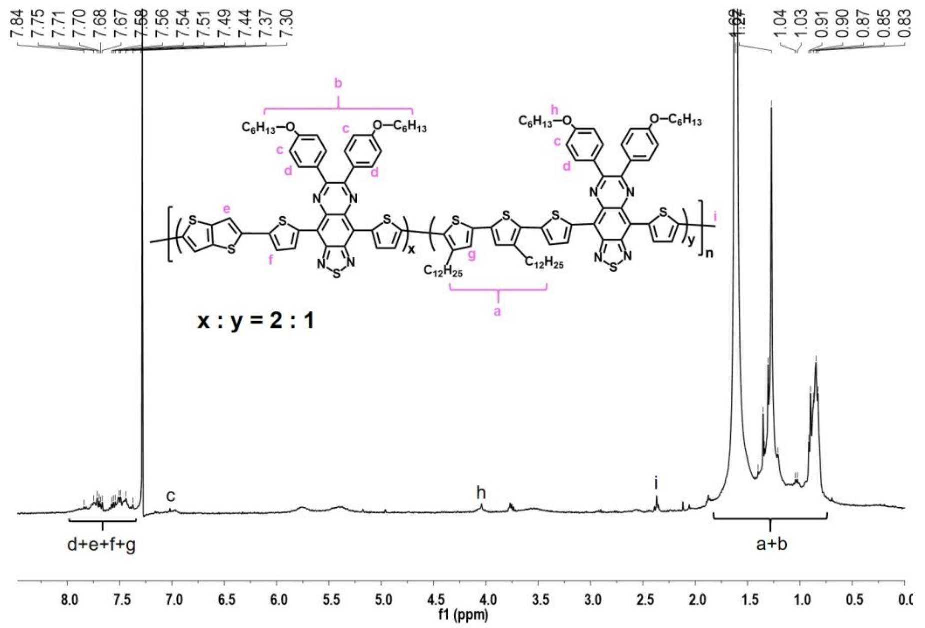

Figure S1. ^{1}H NMR spectrum of NIR-II polymer SP1 in $CDCl_3$.

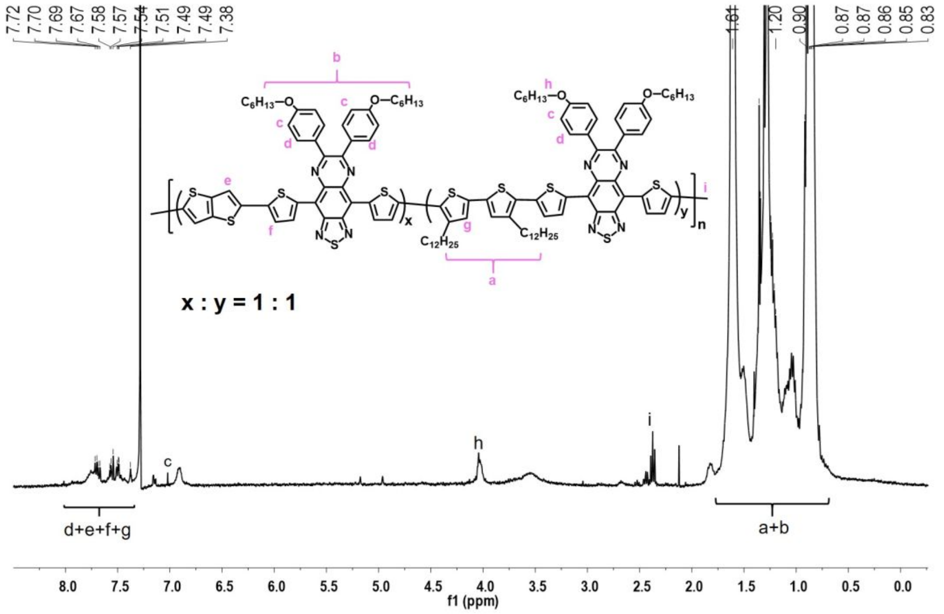

Figure S2. ^{1}H NMR spectrum of NIR-II polymer SP2 in $CDCl_3$.

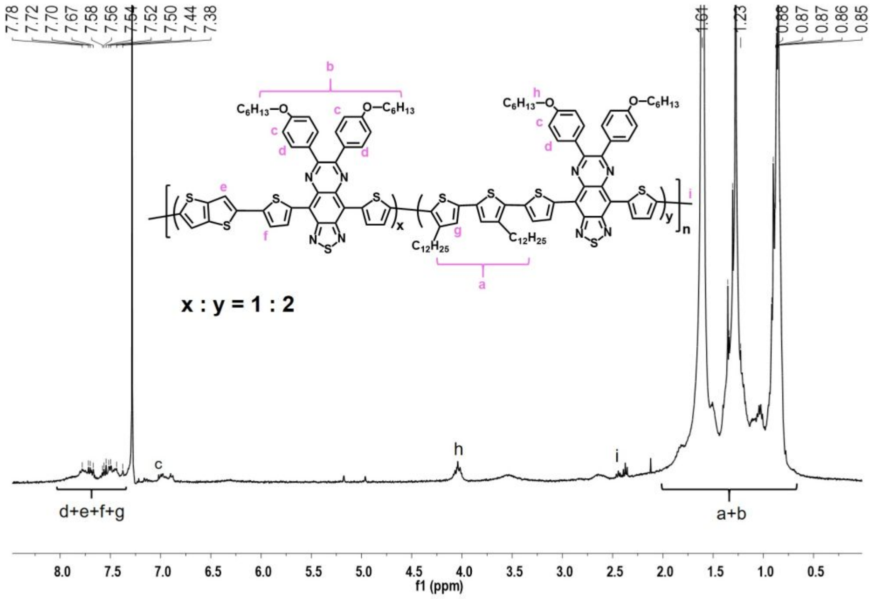

Figure S3. ^{1}H NMR spectrum of NIR-II polymer SP3 in $CDCl_3$.

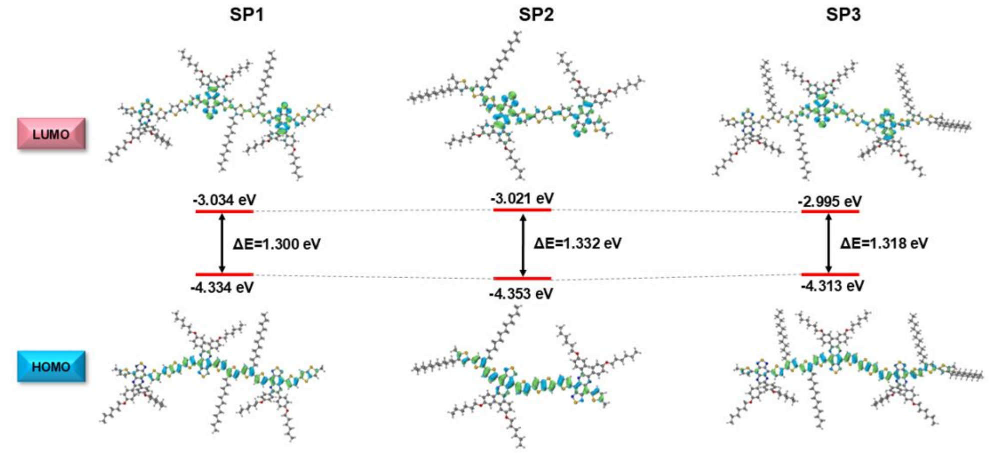

Figure S4. HOMO-LUMO distributions of SPs by DFT calculations at the B3LYP 6-31G(d) level.

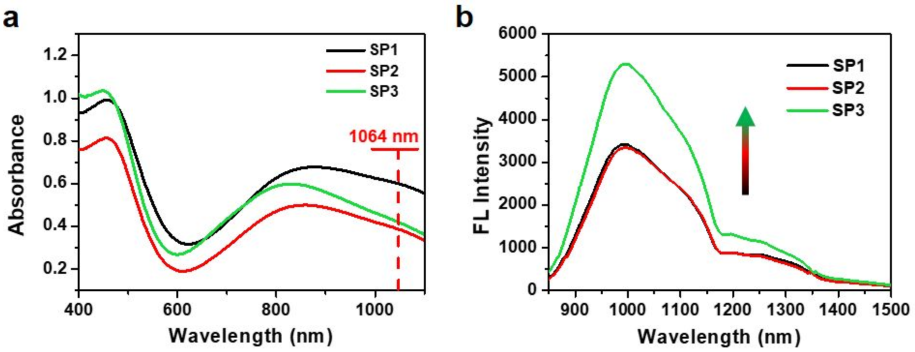

Figure S5. (a) UV-vis absorption spectra and (b) fluorescence emission spectra of SPs with the same mass concentration (50 µg/mL) in tetrahydrofuran (THF).

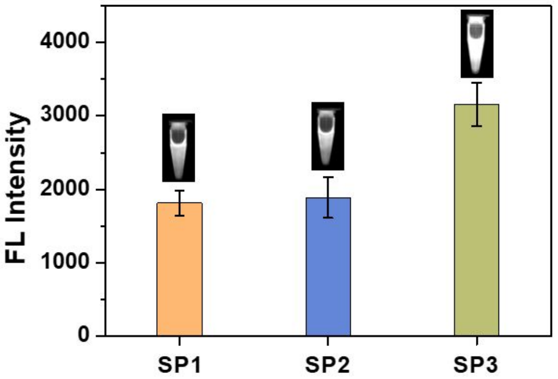

Figure S6. NIR-II fluorescence images (1075 nm long-pass filter) and quantified mean intensity of SPs with the same mass concentration (50 µg/mL) in tetrahydrofuran (THF).

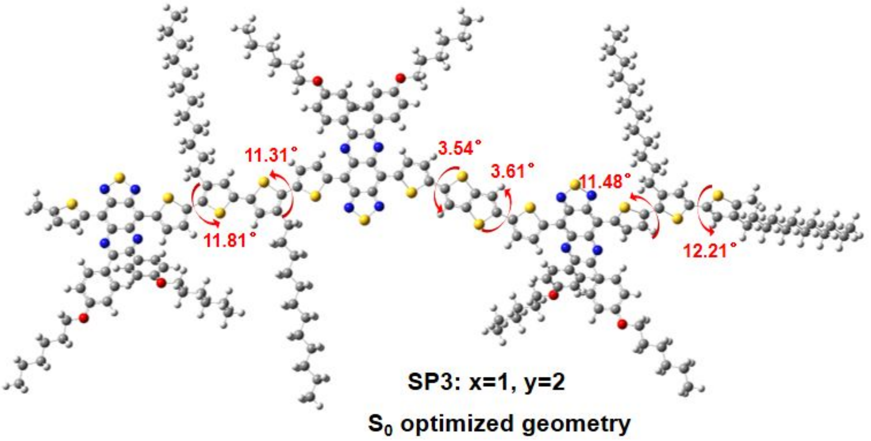

Figure S7. The optimized S_0 geometry of SP3.

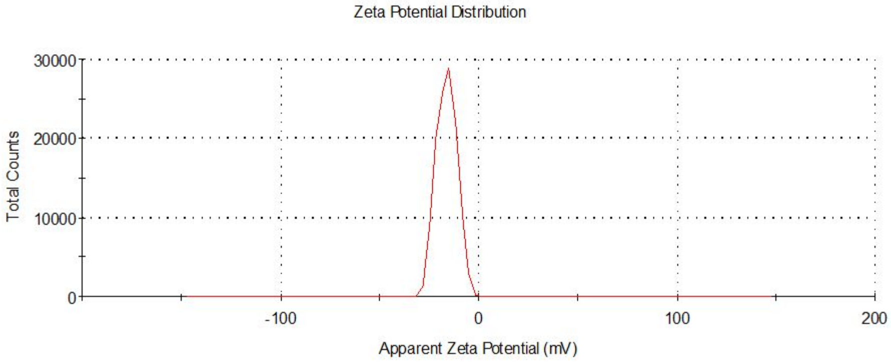

Figure S8. Zeta potential of PSI NPs.

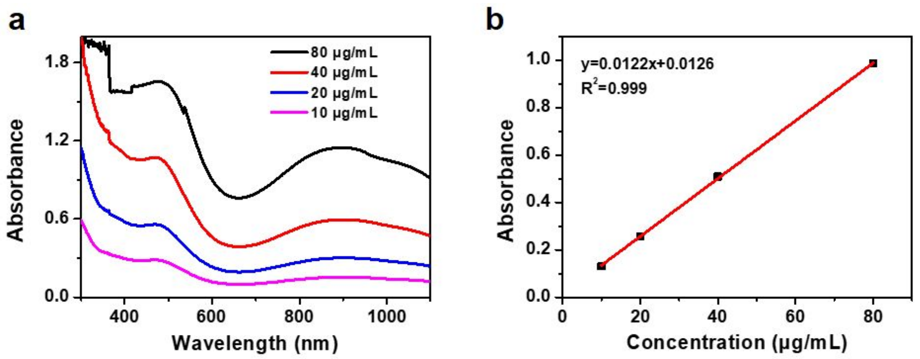

Figure S9. (a) Absorption spectra of PSI NPs with different polymer concentrations. (b) The linear relationship between the absorption at 1064 nm and concentration.

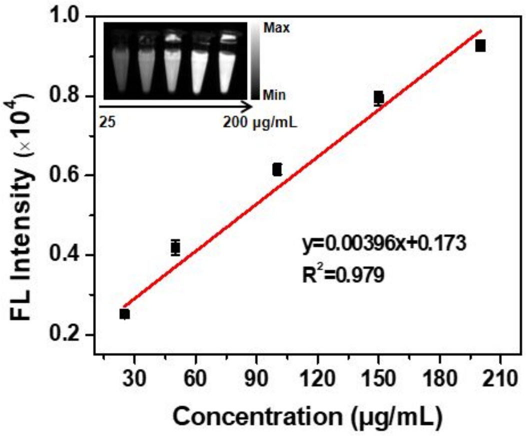

Figure S10. NIR-II fluorescence imaging and fluorescence intensity analysis of PSI NPs aqueous solutions with various concentrations.

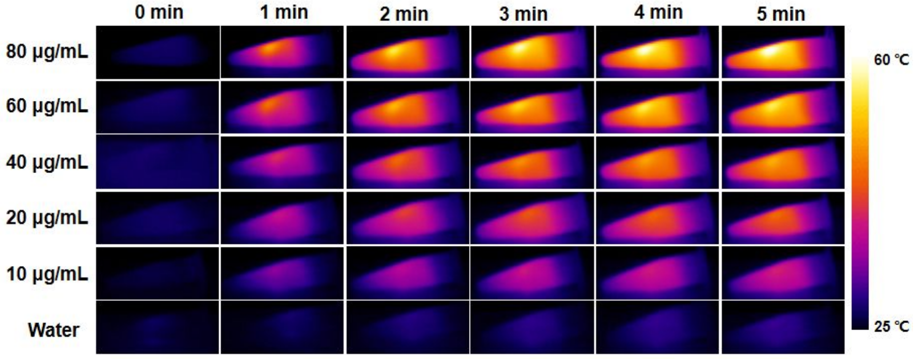

Figure S11. Photothermal images of PSI NPs aqueous solutions with various concentrations over time under 1064 nm laser irradiation (1 W/cm^2).

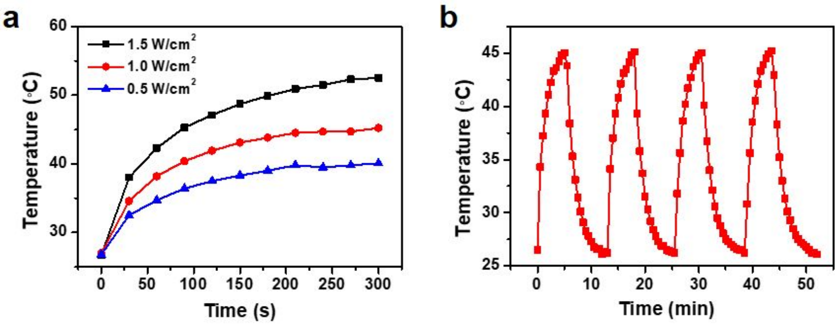

Figure S12. (a) Laser power density dependent temperature changes of PSI NPs solution. (b) Photothermal changes under four laser on-off cycles.

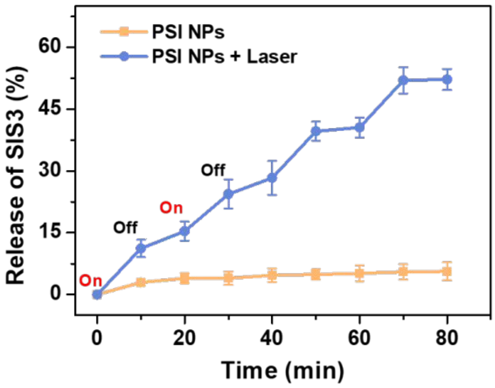

Figure S13. Release of SIS3 from PSI NPs with or without 1064 nm laser irradiation.

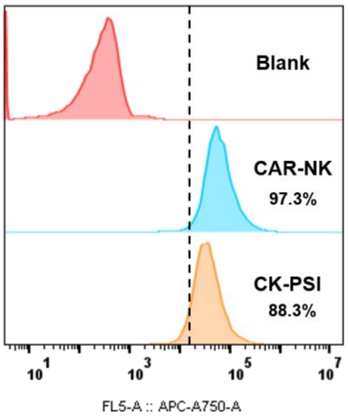

Figure S14. The binding affinity of CAR-NK cells and CK-PSI against B7H3 antigen.

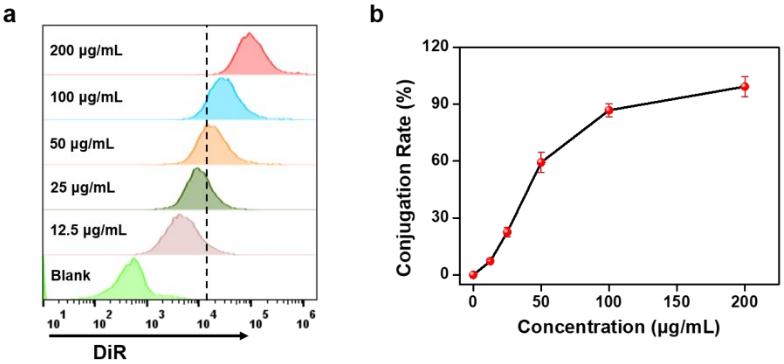

Figure S15. Quantitative analysis of the conjugation efficiency of PSI NPs with various concentrations towards CAR-NK cells.

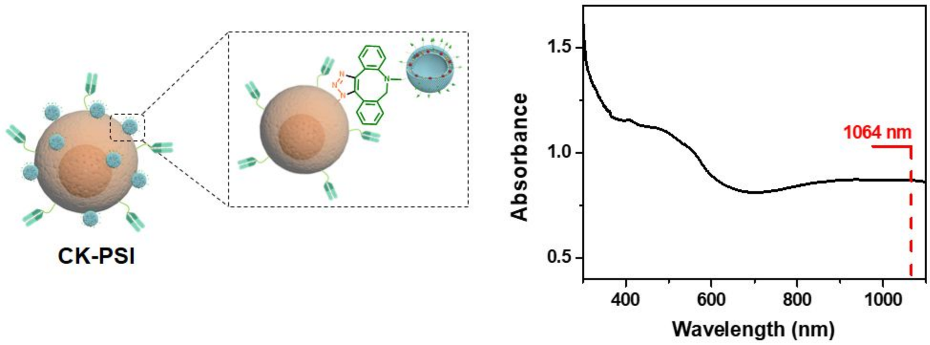

Figure S16. UV-vis absorption spectrum of CK-PSI solution.

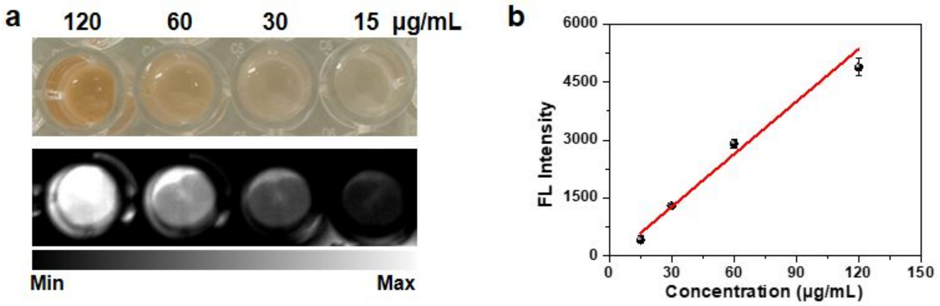

Figure S17. (a) NIR-II fluorescence imaging and (b) fluorescence intensity analysis of CK-PSI solution with various polymer concentrations.

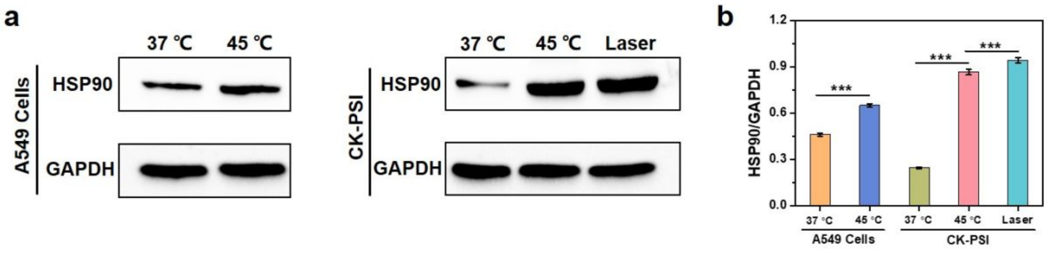

Figure S18. (a) Western blot assay and (b) determination of HSP90 protein expression in A549 cells and CK-PSI after various treatments. ***$p < 0.001$.

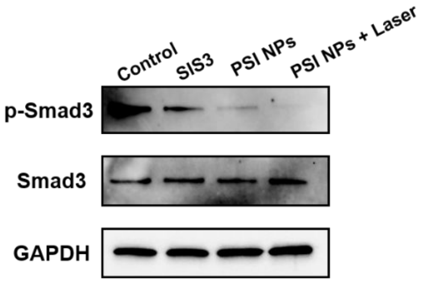

Figure S19. Western blot assay of p-Smad3 protein expression in CAFs after various treatments.

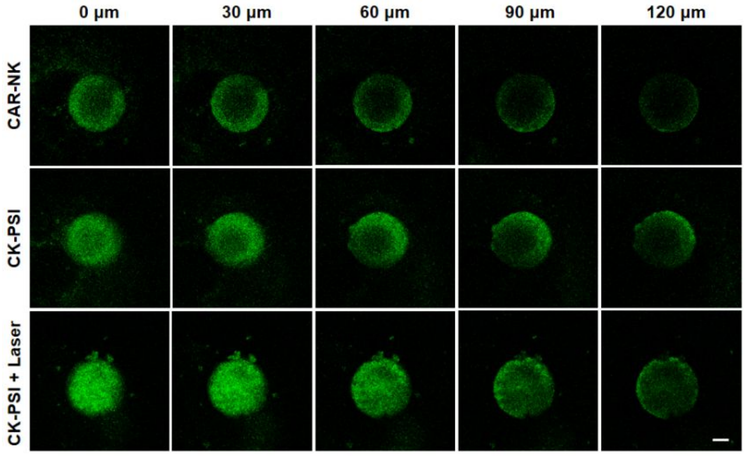

Figure S20. Fluorescence images of A549 3D tumor spheroids at various depths after incubated with Zs-Green modified CAR-NK cells and CK-PSI with or without 1064 nm laser irradiation. Scale bar: 200 μm.

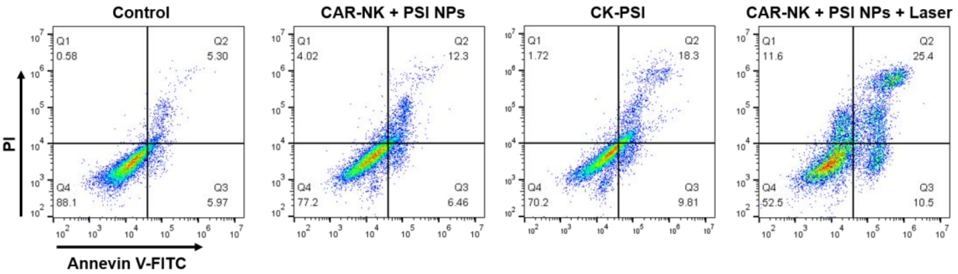

Figure S21. Flow cytometric analysis results of A549 cell apoptosis after various treatments.

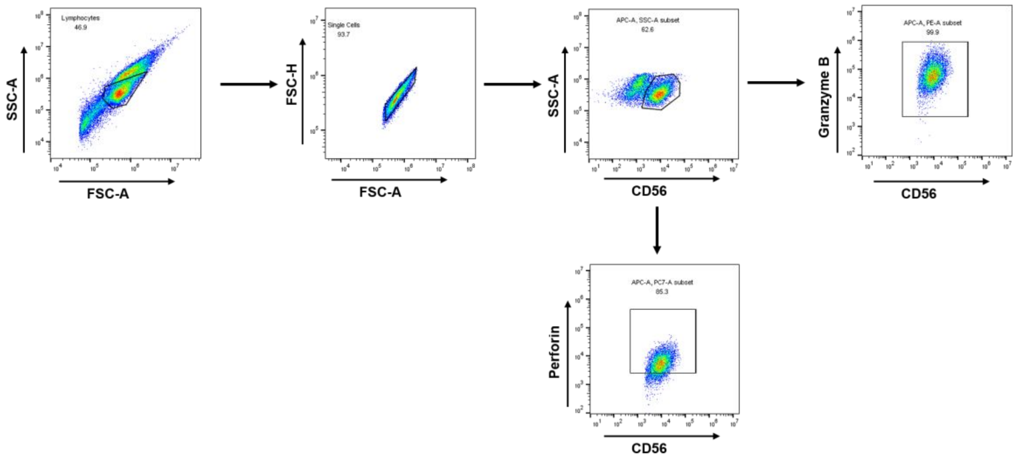

Figure S22. Gating strategies for flow cytometry analysis of granzyme B and perforin secreted by CAR-NK cells after various treatments.

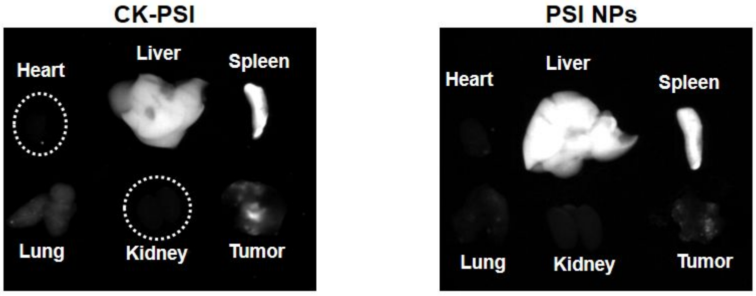

Figure S23. NIR-II fluorescence images of tumors and major organs at 48 h post-injection.

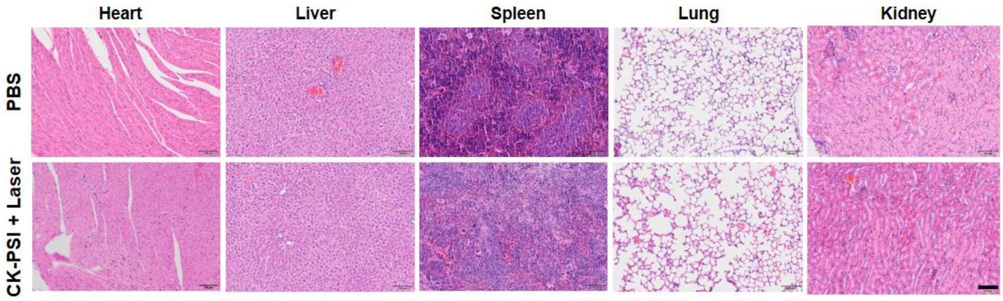

Figure S24. H&E staining images of major organs (heart, liver, spleen, lung and kidney) of A549 tumor-bearing mice with different treatments. Scale bar: 100 μm.

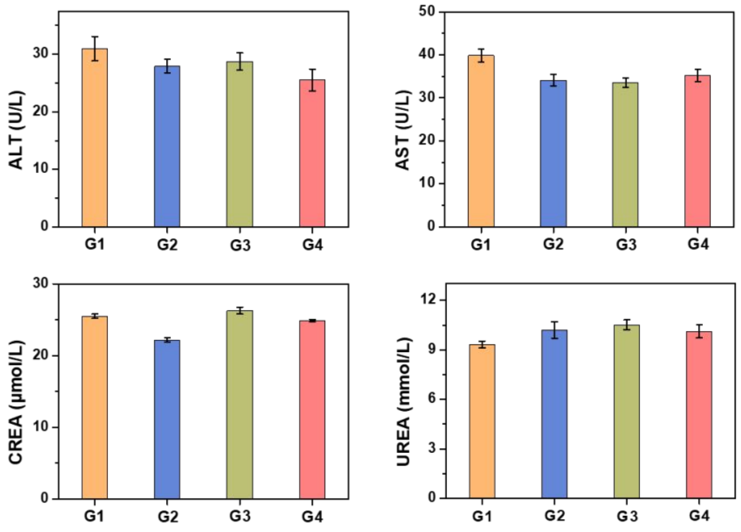

Figure S25. The levels of serum biochemical markers after various treatments, including alanine aminotransferase (ALT), aspartate aminotransferase (AST), creatinine (CREA) and UREA. G1: PBS, G2: CAR-NK, G3: CK-PSI, G4: CK-PSI + Laser.

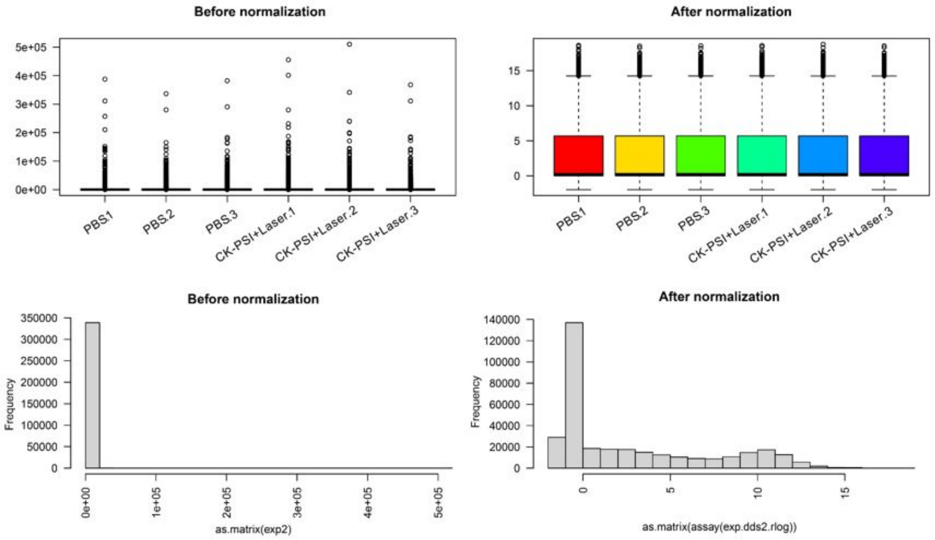

Figure S26. The normalization of gene data using the VST (variance stabilizing transformations) method in DESeq2 before further analysis.

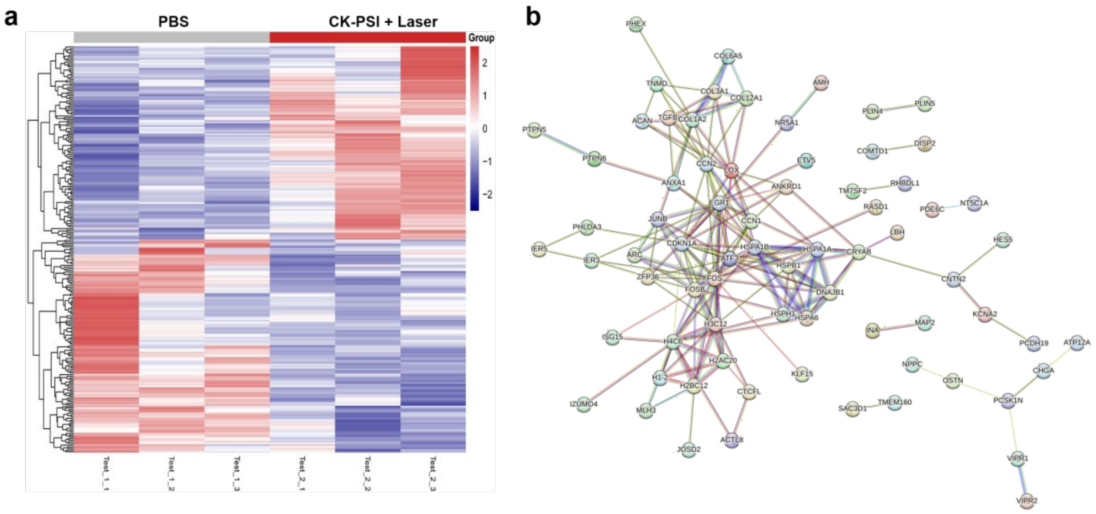

Figure S27. (a) Heat map and (b) protein-protein interaction (PPI) network of the DEGs between PBS group and CK-PSI + Laser group.

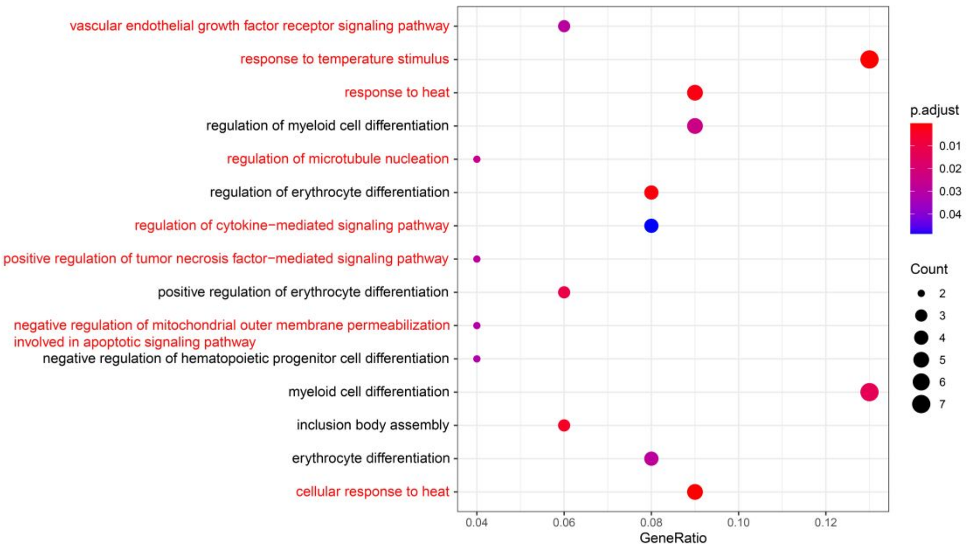

Figure S28. Bubble diagram of GO enrichment analysis of up-regulated DEGs after CK-PSI + Laser treatment.

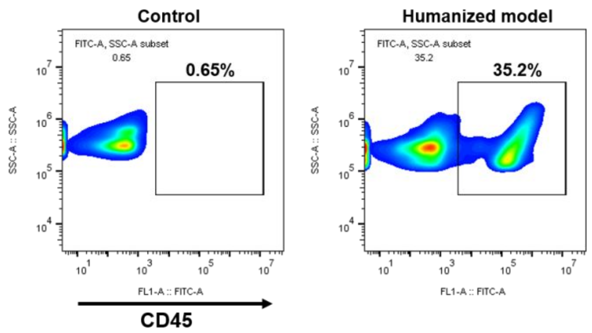

Figure S29. Flow cytometry analysis of immune cells in the peripheral blood of NSG mice two weeks after injection of PBMC.

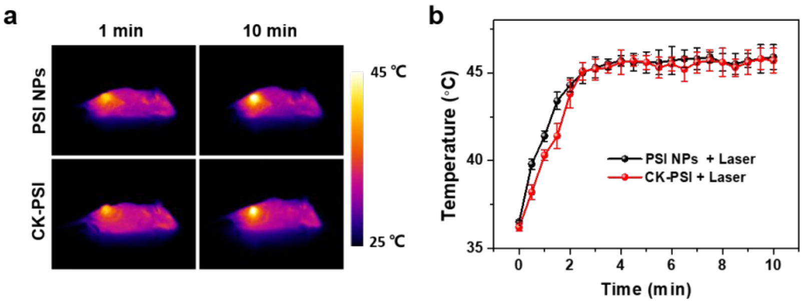

Figure S30. (a) Infrared thermal images and (b) tumor temperature changes of mice in the PSI NPs and CK-PSI injected groups under 1064 nm laser irradiation.

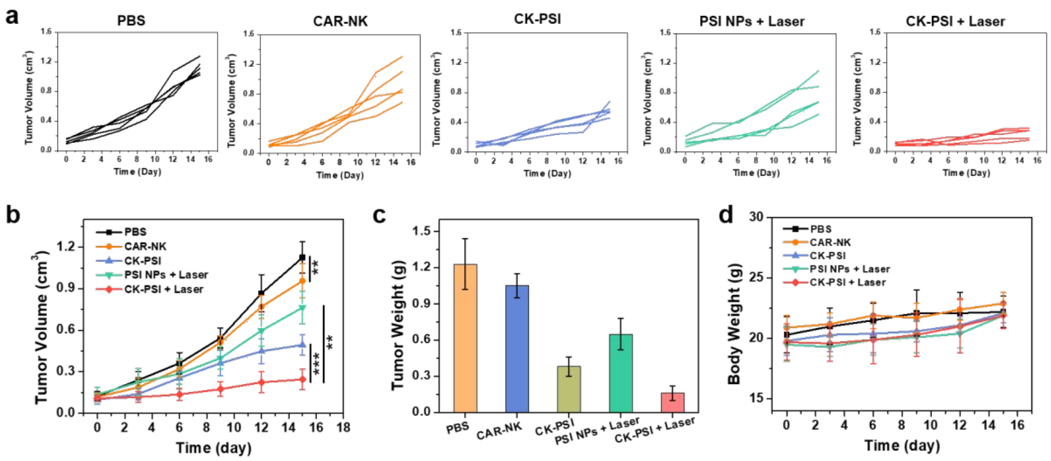

Figure S31. (a) (b) Tumor growth curves of humanized mice after different treatments. (c) Tumor weight after 15 days of treatment. (d) Changes of mouse body weight during the treatment period.

Table S1. GPC results of the synthesized SPs (SP1, SP2 and SP3).

Sample	Mn	Mw	PDI
SP1	58583	77479	1.323
SP2	56600	80519	1.423
SP3	48165	63246	1.313

Reference

(1) Yang, S.; Cao, B. H.; Zhou, G. Y.; Zhu, L. P.; Wang, L.; Zhang, L.; Kwok, H. F.; Zhang, Z. F.; Zhao, Q. Targeting B7-H3 Immune Checkpoint With Chimeric Antigen Receptor-Engineered Natural Killer Cells Exhibits Potent Cytotoxicity Against Non-Small Cell Lung Cancer. *Front. Pharmacol.* **2020**, *11*, 1089.

(2) Huang, S. G.; Xing, F. Q.; Dai, Y. E.; Zhang, Z. M.; Zhou, G. Y.; Yang, S.; Liu, Y. C.; Yuan, Z.; Luo, K. Q.; Ying, T. L.; et al. Navigating chimeric antigen receptor-engineered natural killer cells as drug carriers via three-dimensional mapping of the tumor microenvironment. *J. Controlled Release* **2023**, *362*, 524-535.

(3) Qiu, Z. W.; Zhong, Y. T.; Lu, Z. M.; Yan, N.; Kong, R. J.; Huang, J. Q.; Li, Z. F.; Nie, J. M.; Li, R. Q.; Cheng, H. Breaking Physical Barrier of Fibrotic Breast Cancer for Photodynamic Immunotherapy by Remodeling Tumor Extracellular Matrix and Reprogramming Cancer-Associated Fibroblasts. *ACS Nano* **2024**, *18* (13), 9713-9735.
